# Combinatorial Properties and Recognition of Unit Square Visibility Graphs

**DOI:** 10.1007/s00454-022-00414-8

**Published:** 2023-03-22

**Authors:** Katrin Casel, Henning Fernau, Alexander Grigoriev, Markus L. Schmid, Sue Whitesides

**Affiliations:** 1 Institut für Informatik, Humboldt-Universität zu Berlin, Unter den Linden 6, 10099 Berlin, Germany; 2 Fachbereich 4 – Abteilung Informatikwissenschaften, Universität Trier, 54286 Trier, Germany; 3 School of Business and Economics, Maastricht University, P.O.Box 616, 6200 MD Maastricht, The Netherlands; 4 Department of Computer Science, University of Victoria, P.O.Box 1700, STN CSC, Victoria, BC V8W 2Y2 Canada

**Keywords:** Geometric graph classes, Graph recognition, Visibility graphs, Visibility layout, ${\textsf{N}}{\textsf{P}}$-completeness, 68R10, 05C10, 05C62

## Abstract

Unit square visibility graphs (USV) are described by axis-parallel visibility between unit squares placed in the plane. If the squares are required to be placed on integer grid coordinates, then USV become unit square grid visibility graphs (USGV), an alternative characterisation of the well-known rectilinear graphs. We extend known combinatorial results for USGV and we show that, in the weak case (i.e., visibilities do not necessarily translate into edges of the represented combinatorial graph), the area minimisation variant of their recognition problem is ${{\,\mathrm{{\textsf{N}}{\textsf{P}}}\,}}$-hard. We also provide combinatorial insights with respect to USV, and as our main result, we prove their recognition problem to be ${{\,\mathrm{{\textsf{N}}{\textsf{P}}}\,}}$-hard, which settles an open question.

## Introduction

A visibility representation of a graph *G* is a set ${\mathcal {R}} = \{R_i\,|\,1 \le i \le n\}$ of geometric objects (e.g., bars, rectangles, etc.) along with some kind of geometric visibility relation $\sim$ over ${\mathcal {R}}$ (e.g., axis-parallel visibility), such that $G = (\{v_i \,|\,1 \le i \le n\}, \{\{v_i, v_j\} \mid R_i\,{\sim }\,R_j\})$. In this work, we focus on rectangle visibility graphs, which are represented by axis aligned rectangles in the plane and vertical and horizontal axis parallel visibility between them. In particular, we consider the more restricted variant of *unit square visibility graphs* (see [12]), and, in addition, we also consider the case where the unit squares are placed on an integer grid (an alternative characterisation of the well-known class of graphs with rectilinear drawings).

The study of visibility representations is of interest, both for applications and for graph classes, and has remained an active research area^1^ mainly because axis-aligned visibilities give rise to graph and network visualizations that satisfy good readability criteria: straight edges, and edges that cross only at right angles. These properties are highly desirable in the design of layouts of circuits and communication paths. Indeed, the study of graphs arising from vertical visibilities among disjoint, horizontal line segments (“bars”) in the plane originated during the 1980’s in the context of VLSI design problems; see [18, 32, 33].

Because bar visibility graphs are necessarily planar, this model has been extended in various ways in order to represent larger classes of graphs. Such extensions include new definitions of visibility (e.g., sight lines that may penetrate up to *k* bars [13] or other geometric objects [4]), vertex representations by other objects (e.g., rectangles, L-shapes [20], ortho-polygons [6], and sets of up to *t* bars [25]), extensions to higher dimensional objects (see, e.g., [8] for visibility representation in 3D by axis aligned horizontal rectangles with vertical visibilities, or [21], which studies visibility representations by unit squares floating parallel to the *x*, *y*-plane and lines of sight that are parallel to the *z*-axis). The desire for polysemy, that is, the expression of more than one graph by means of one underlying set of objects, has also provided impetus in the study of visibility representations (see for example [6, 20, 31]).

Rectangle visibility graphs have the attractive property, for visualization purposes, that they yield right angle crossing drawings (RAC graphs (see [17]), which are graphs with poly-line drawings such that any two crossing segments are orthogonal), which have seen considerable interest in the graph drawing community. Unit square graphs form a subfamily of L-visibility graphs (see [20]) and their grid variant a subfamily of RACs with no bends (note that RAC recognition for 0-bends is ${{\,\mathrm{{\textsf{N}}{\textsf{P}}}\,}}$-hard [2]).

Using visibilities among objects is but one example of the use of binary geometric relations for this purpose; other geometric relations include intersection relations (e.g., of strings or straight line segments in the plane, of boxes in arbitrary dimension), proximity relations (e.g., of points in the plane), and contact relations. In the literature, for the resulting graph classes, combinatorial aspects, relationships to other graph classes, as well as computational aspects are studied (see [22] for a survey focusing on contact representations of rectangles).

Finally, we note that visibility properties among sets of objects have been studied in a number of contexts, including motion planning and computer graphics. In [29] it is proposed to find shortest paths for mobile robots moving in a cluttered environment by looking for shortest paths in the visibility graph of the points located at the vertices of polygonal obstacles. This led to a search for fast algorithms to compute visibility graphs of polygons, as well as to a search for finding shortest paths without computing the entire visibility graph.

### Our Contribution

With respect to unit square grid visibility graphs, we extend the known combinatorial results (since unit square grid visibility graphs are equivalent to rectilinear graphs, many such results already exist), in particular, with respect to planarity and characterisations, and we show that the area minimisation variant of the recognition problem (i.e., deciding whether a given graph can be represented by a layout within some given height and width bounds) for *weak* (see Sect. 2) unit square grid visibility graphs is ${{\,\mathrm{{\textsf{N}}{\textsf{P}}}\,}}$-hard. From our reduction, we are also able to conclude hardness for some other variants of the recognition problem.

For unit square visibility graphs (i.e., the case where the positions of the unit squares are not restricted to integer coordinates), we also prove some combinatorial results (thus, we extend the investigations initiated by [12]). As our main result, we settle the open question regarding the complexity of the recognition problem for this graph class, by proving its ${{\,\mathrm{{\textsf{N}}{\textsf{P}}}\,}}$-hardness. This requires a reduction that is highly non-trivial on a technical level with the main difficulty to identify graph structures that can be shown to be representable by unit square layouts in a unique way to gain sufficient control for designing suitable gadgets.

### Organisation of the Paper

In Sect. 2, we formally define the considered classes of visibility graphs and we recall some more classical geometric graph classes that are similar to the unit square grid visibility graphs. Sections 3 and 4 are then devoted to the grid variant and the non-grid variant, respectively, of unit square visibility graphs. More precisely, we investigate combinatorial properties of unit square grid visibility graphs and the area minimisation variant of the recognition problem for weak unit square grid visibility graphs in Sects. 3.1 and 3.2, respectively. Section 4 considers unit square visibility graphs, starts with combinatorial results and the hardness of the recognition problem is shown in Sect. 4.1, which, due to the intricacy of the whole construction, is further divided in a first part with some preliminaries and general ideas (Sect. 4.1.1), followed by the formal definition of the reduction (Sect. 4.1.2) and the actual proof that the reduction is correct (Sect. 4.1.3).

## Preliminaries

A *visibility layout*, or simply *layout*, is a set ${\mathcal {R}} = \{R_i \;|\; 1 \le i \le n\}$ with $n \in {\mathbb {N}}$, where $R_i$ are closed axis-parallel rectangles in the plane; the *position* of such a rectangle is the coordinate of its lower left corner. We further ask that any two different rectangles intersect in at most one point, i.e., touching corners are allowed (we shall discuss this decision, and more generally the issue of touching corners or borders with respect to rectangle visibility graphs, in Sect. 2.1 further below).

For every $R_i, R_j \in {\mathcal {R}}$ with $R_i \ne R_j$, a closed non-degenerate axis-parallel rectangle *S* (i.e., a non-empty closed rectangle that is not a line segment) is a *visibility rectangle for *$R_i$ *and* $R_j$ if one side of *S* is contained in $R_i$ and the opposite side in $R_j$. In particular, corner-touching does not enable visibility. We define $R_i {{\,\mathrm{\rightarrow }\,}}_{{\mathcal {R}}} R_j$ ($R_i {{\,\mathrm{\downarrow }\,}}_{{\mathcal {R}}} R_j$), if there is a visibility rectangle *S* for $R_i$ and $R_j$, such that the left side (upper side) of *S* is contained in $R_i$, the right side (lower side) of *S* is contained in $R_j$ and $S \cap R_k = \emptyset$, for every $R_k \in {\mathcal {R}} \setminus \{R_i, R_j\}$. Let ${{\,\mathrm{\leftrightarrow }\,}}_{{\mathcal {R}}}$ and ${{\,\mathrm{\updownarrow }\,}}_{{\mathcal {R}}}$ be the symmetric closures of ${{\,\mathrm{\rightarrow }\,}}_{{\mathcal {R}}}$ and ${{\,\mathrm{\downarrow }\,}}_{{\mathcal {R}}}$, respectively. Finally, $R_i {{\,\mathrm{\sim }\,}}_{{\mathcal {R}}} R_j$ if $R_i {{\,\mathrm{\leftrightarrow }\,}}_{{\mathcal {R}}} R_j$ or $R_i {{\,\mathrm{\updownarrow }\,}}_{{\mathcal {R}}} R_j$ (${{\,\mathrm{\sim }\,}}_{{\mathcal {R}}}$ is the *visibility relation* (*with respect to* ${\mathcal {R}}$)). If the layout ${\mathcal {R}}$ is clear from the context or negligible, we drop the subscript ${\mathcal {R}}$. We denote $R_i {{\,\mathrm{\sim }\,}}R_j$, $R_i {{\,\mathrm{\leftrightarrow }\,}}R_j$, and $R_i {{\,\mathrm{\rightarrow }\,}}R_j$ also as $R_i$ *sees* $R_j$, $R_i$ *horizontally sees* $R_j$, and $R_i$ *sees* $R_j$ *from the left*, respectively, and analogous terminology applies to vertical visibilities. For $S,T\subseteq {\mathcal {R}}$, we use $S {{\,\mathrm{\rightarrow }\,}}_{{\mathcal {R}}} T$ to mean $R {{\,\mathrm{\rightarrow }\,}}_{{\mathcal {R}}} R'$ for all $R \in S$ and $R'\in T$.

A layout ${\mathcal {R}} = \{R_i\,|\,1 \le i \le n\}$ *represents* the undirected graph ${{\,\mathrm{{\textsf{G}}}\,}}({\mathcal {R}}) = (\{v_i \,|\,1 \le i \le n\}, \{\{v_i, v_j\}\mid 1 \le i, j \le n,\, R_i {{\,\mathrm{\sim }\,}}R_j\})$, which is then called a *visibility graph*, and the class of visibility graphs is denoted by ${{\,\mathrm{{\textsf{V}}}\,}}$. A graph is a *weak* visibility graph, if it can be obtained from a visibility graph by deleting some edges and the corresponding class of graphs is denoted by ${{\,\mathrm{{\textsf{V}}}\,}}_{{{\,\mathrm{{\textsf{w}}}\,}}}$. As a convention, for a visibility graph $G = (V, E)$ and a layout representing it we denote by $R_v$ the rectangle for $v\in V$ and define $R_{V'}=\{R_x\,|\,x \in V'\}$ for every $V' \subseteq V$. We call layouts ${\mathcal {R}}_1$ and ${\mathcal {R}}_2$ *isomorphic* if ${{\,\mathrm{{\textsf{G}}}\,}}({\mathcal {R}}_1)$ and ${{\,\mathrm{{\textsf{G}}}\,}}({\mathcal {R}}_2)$ are isomorphic. Furthermore, we call ${\mathcal {R}}_1$ and ${\mathcal {R}}_2$ V *-isomorphic* if, for some $x \in \{{{\,\mathrm{\rightarrow }\,}}_{{\mathcal {R}}_1}, {{\,\mathrm{\rightarrow }\,}}^{-1}_{{\mathcal {R}}_1}\}$ and $y \in \{{{\,\mathrm{\downarrow }\,}}_{{\mathcal {R}}_1}, {{\,\mathrm{\downarrow }\,}}^{-1}_{{\mathcal {R}}_1}\}$, the relational structure $({\mathcal {R}}_1, {{\,\mathrm{\rightarrow }\,}}_{{\mathcal {R}}_1}, {{\,\mathrm{\downarrow }\,}}_{{\mathcal {R}}_1})$ is isomorphic to $({\mathcal {R}}_2, x, y)$ or $({\mathcal {R}}_2, y, x)$.^2^

*Unit square visibility graphs* (${{\,\mathrm{\textsf{USV}}\,}}$) and *unit square grid visibility graphs* (${{\,\mathrm{\mathrm {\textsf {USGV}}}\,}}$) are represented by *unit square layouts* ${\mathcal {R}}$, where every $R\in {\mathcal {R}}$ is a unit square, and *unit square grid layouts*, where additionally the position of every *R* is from ${\mathbb {N}}\times {\mathbb {N}}$. Note that in the grid case, if a unit square is positioned at (*x*, *y*), then there is no other unit square on coordinates (*x*, *y*), and no unit square on coordinates $(x+1, y)$, $(x, y+1)$, $(x, y-1)$, or $(x-1, y-1)$.

### Observation 2.1

If $R_u \downarrow R_v$ is in a USGV representation, then $R_w \downarrow R_v$, $R_u\downarrow R_w$, $R_w \uparrow R_u$, and $R_v \uparrow R_w$ are not in the representation for any $R_w \ne R_u, R_v$.

The weak classes ${{\,\mathrm{\textsf{USV}}\,}}_{{{\,\mathrm{{\textsf{w}}}\,}}}$ and ${{\,\mathrm{\mathrm {\textsf {USGV}}}\,}}_{{{\,\mathrm{{\textsf{w}}}\,}}}$ are defined accordingly.

For a graph $G = (V, E)$, *N*(*v*) is the *neighbourhood* of $v \in V$, $\vec {E}$ denotes an oriented version of *E*, i.e., $E = \{\{u, v\} \mid (u, v) \in \vec {E}\}$, and $f:\vec {E}\rightarrow E$, $(u,v)\mapsto \{u,v\}$, is a bijection. Let ${{\,\mathrm{{\textsf{L}}}\,}}, {{\,\mathrm{{\textsf{R}}}\,}}$ and ${{\,\mathrm{{\textsf{D}}}\,}}, {{\,\mathrm{{\textsf{U}}}\,}}$ be pairs of complementary values (for $X \in \{{{\,\mathrm{{\textsf{L}}}\,}}, {{\,\mathrm{{\textsf{R}}}\,}}, {{\,\mathrm{{\textsf{D}}}\,}}, {{\,\mathrm{{\textsf{U}}}\,}}\}$, ${\overline{X}}$ denotes its complement). An ${{\,\mathrm{\textsf{LRDU}}\,}}$-*restriction* (for *G*) is a labelling $\sigma :\vec {E} \rightarrow \{{{\,\mathrm{{\textsf{L}}}\,}}, {{\,\mathrm{{\textsf{R}}}\,}}, {{\,\mathrm{{\textsf{D}}}\,}}, {{\,\mathrm{{\textsf{U}}}\,}}\}$ and it is *valid* if, for every $(u, v) \in \vec {E}$ with $\sigma ((u, v)) = X$ and every $w \in V \setminus \{u, v\}$, $\sigma ((u, w)) \ne X \ne \sigma ((w, v))$, and $\sigma ((v, w)) \ne {\overline{X}} \ne \sigma ((w, u))$. Obviously, ${{\,\mathrm{\textsf{LRDU}}\,}}$-restrictions are only a reasonable concept for graphs with maximum degree 4. A unit square grid visibility layout *satisfies* an ${{\,\mathrm{\textsf{LRDU}}\,}}$-restriction $\sigma$ if $\sigma ((u, v)) = {{\,\mathrm{{\textsf{L}}}\,}}$ implies $R_{v} {{\,\mathrm{\rightarrow }\,}}R_{u}$, $\sigma ((u, v)) = {{\,\mathrm{{\textsf{R}}}\,}}$ implies $R_{u} {{\,\mathrm{\rightarrow }\,}}R_{v}$, $\sigma ((u, v)) = {{\,\mathrm{{\textsf{D}}}\,}}$ implies $R_{u} {{\,\mathrm{\downarrow }\,}}R_{v}$ and $\sigma ((u, v)) = {{\,\mathrm{{\textsf{U}}}\,}}$ implies $R_{v} {{\,\mathrm{\downarrow }\,}} R_{u}$. An ${{\,\mathrm{{\textsf{H}}{\textsf{V}}}\,}}$-*restriction* (for *G*) is a labelling $\sigma :E \rightarrow \{{{\,\mathrm{{\textsf{H}}}\,}}, {{\,\mathrm{{\textsf{V}}}\,}}\}$ and it is *valid* if, for every $u \in V$ at most two incident edges are labeled ${{\,\mathrm{{\textsf{H}}}\,}}$ and at most two incident edges are labeled ${{\,\mathrm{{\textsf{V}}}\,}}$. A unit square grid visibility layout *satisfies* an ${{\,\mathrm{{\textsf{H}}{\textsf{V}}}\,}}$-restriction $\sigma$ if $\sigma (\{u, v\}) = {{\,\mathrm{{\textsf{H}}}\,}}$ implies $R_{v} {{\,\mathrm{\leftrightarrow }\,}}R_{u}$ and $\sigma (\{u, v\}) = {{\,\mathrm{{\textsf{V}}}\,}}$ implies $R_{v} {{\,\mathrm{\updownarrow }\,}} R_{u}$.

For any set ${\mathfrak {G}}$ of undirected graphs, we define the following problem:

Recognition for $${\mathfrak {G}}$$ ($${{\,\mathrm{\textsc {Rec}}\,}}({\mathfrak {G}}))$$

*Instance*: Undirected graph *G*.

*Question*: $$G \in {\mathfrak {G}}$$?

In the following, we shall consider the problems ${{\,\mathrm{\textsc {Rec}}\,}}({{\,\mathrm{\mathrm {\textsf {USGV}}}\,}})$ and ${{\,\mathrm{\textsc {Rec}}\,}}({{\,\mathrm{\textsf{USV}}\,}})$.

We briefly recall some established geometric graph representations relevant to this work. A *rectilinear drawing* (see [19, 28]) of a graph $G = (V, E)$ is a pair of mappings $x, y :V \rightarrow {\mathbb {Z}}$, where, for every $v \in V$, *x*(*v*) and *y*(*v*) represent the *x*- and *y*-coordinates of *v* on the grid and, for every edge $\{u, v\} \in E$, (*x*(*u*), *y*(*u*)) and (*x*(*v*), *y*(*v*)) are the endpoints of a horizontal or vertical line segment that does not contain any (*x*(*w*), *y*(*w*)) with $w \in V \setminus \{u, v\}$. A graph is called a *rectilinear graph* if it has a rectilinear drawing. A graph has *resolution* ${2\pi }/{d}$ if it has a drawing in which the degree of the angle between any two edges incident to a common vertex is at least ${2\pi }/{d}$. We call such graphs *resolution-*$({2\pi }/{d})$ *graphs* and are mainly interested in the case $d = 4$, see [23]. Planar graphs with resolution at least ${\pi }/{2}$ are rectilinear, see [7]. A *bendless right angle crossing* (BRAC) *drawing* of a graph is a straight-line drawing in which every crossing of two edges is at right angles.^3^ Note that in a BRAC drawing or a resolution-$(2\pi /{4})$ drawing, edges are not necessarily axis-parallel (as is the case for visibility layouts and rectilinear drawings). A graph is called a *BRAC graph* if it has a BRAC drawing.

### A Remark on Corner- and Border-Intersections of Rectangles

In the literature on rectangle visibility graphs, it is usually required that rectangles are pairwise disjoint, but it is not always made precise what this means. In particular, it is common to allow rectangles to intersect in corners (see [12]), or to allow even overlapping boundaries (see [14]).^4^

Since our paper mainly extends the work initiated by [12], we choose to adopt the respective definitions, i.e., we allow two squares to overlap in at most one point, which means that they can only intersect in at most one corner. It should be noted, however, that these seemingly small differences, i.e., whether we allow or disallow rectangles to intersect in corners or borders, lead to different graph classes.

**Fig. 1:**
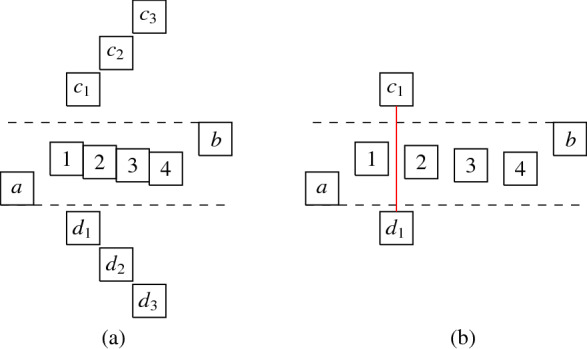
Example illustrating that there are graphs that can only be represented with layouts that allow intersection of borders

More precisely, the three versions (I) “no intersection”, (II) “corner-touching”, and (III) “border intersection” yield a strict hierarchy of graph classes. For example, Fig. 5(b) shows a layout for the complete bipartite graph $K_{2,6}$ that has unit squares with intersecting corners (type (II)), but we cannot represent $K_{2,6}$ if we require strictly non-intersecting unit squares (type (I)). Moreover, consider a graph with vertices $\{a, b, i, c_j, d_j \mid 1 \le i \le k, \,1 \le j \le k-1\}$ and with edges such that $1, 2, \ldots , k$ forms a path in this order with all vertices adjacent to both *a* and *b*, and, for every $1 \le i \le k-1$, both $c_i$ and $d_i$ are adjacent to both vertices *i* and $i+1$. Figure 1(a) shows how this graph (for $k=4$) can be represented by a unit square visibility layout with border intersections (type (III)). However, Fig. 1(b) illustrates that if unit squares are not allowed to have touching borders (type (I) or (II)), then we necessarily have to create some unwanted visibilities.

## Unit Square Grid Visibility Graphs

The readability of graph drawings is mainly affected by its *angular resolution* (i.e., the minimum angle formed by consecutive edges incident to a common node) and its *crossing resolution* (angles formed at edge crossings); see the discussion in [1]. In this regard, resolution-$({\pi }/{2})$ graphs and BRAC graphs have an angular resolution and crossing resolution of ${\pi }/{2}$, respectively, while rectilinear drawings and unit square grid visibility layouts force *both* resolutions to be ${\pi }/{2}$.

The question arises of how these classes relate to each other and in this regard, we first note that ${{\,\mathrm{\mathrm {\textsf {USGV}}}\,}}$ and rectilinear graphs coincide. More precisely, a unit square grid layout can be transformed into a rectilinear drawing by replacing every unit square on position (*x*, *y*) by a vertex on position (*x*, *y*) and translate the former visibilities into straight-line segments. Transforming a rectilinear drawing into a unit square grid layout can be done by scaling it first by factor 2 and then replacing each vertex on position (*x*, *y*) by a unit square on position (*x*, *y*) (without scaling, sides or corners of unit squares may overlap). This only results in a *weak* layout, since visibilities may be created that do not correspond to edges in the rectilinear drawing. However, any weak unit square grid visibility graph can be transformed into a unit square grid visibility graph (as formally stated below in Theorem 3.7).

Since all these graphs except the BRAC graphs have maximum degree 4, we only consider degree-4 BRAC graphs. Obviously, resolution-$(\pi /2)$ graphs and degree-4 BRAC graphs are both superclasses of ${{\,\mathrm{\mathrm {\textsf {USGV}}}\,}}$ (and rectilinear graphs). Witnessed by $K_3$, the inclusion in degree-4 BRAC graphs is proper, while the analogous question w.r.t. resolution-$(\pi /2)$ graphs is open. Moreover, $K_3$ is also an example of a degree-4 BRAC graph that is not a resolution-$({\pi }/{2})$ graph; whether there exist resolution-$({\pi }/{2})$ graphs without a BRAC drawing is open (in this regard, note that the characterisation of the complete bipartite graphs with BRAC drawings of [16] shows that all complete bipartite resolution-$(\pi /2)$ graphs also have BRAC drawings (in fact, as can be easily verified, $K_{n, m}$ is a resolution-$({\pi }/{2})$ graph if and only if ($n = 1$ and $m \le 4$) or $n = m = 2$)).

Due to the equivalence of ${{\,\mathrm{\mathrm {\textsf {USGV}}}\,}}$ and rectilinear graphs, results for the latter graph class carry over to the former. In this regard, we first mention that the ${{\,\mathrm{{\textsf{N}}{\textsf{P}}}\,}}$-hardness proof of recognizing resolution-$({\pi }/{2})$ graphs from [23] actually produces drawings with axis-aligned edges; thus, it also applies to rectilinear graphs (a similar reduction (for rectilinear graphs and presented in more detail) is provided in [19]). As shown in [19], the recognition problem for rectilinear graphs can be solved in time ${{\,\textrm{O}\,}}(24^kk^{2k}n)$, where *k* is the number of vertices with degree at least 3. In [28], it is shown that recognition remains ${{\,\mathrm{{\textsf{N}}{\textsf{P}}}\,}}$-hard if we ask whether a drawing exists that satisfies a given ${{\,\mathrm{{\textsf{H}}{\textsf{V}}}\,}}$-restriction^5^ or a drawing that satisfies a given circular order of incident edges. However, checking the existence of a rectilinear drawing satisfying a given ${{\,\mathrm{\textsf{LRDU}}\,}}$-restriction can be done in time ${{\,\textrm{O}\,}}(|E|\,{\cdot }\,|V|)$. Consequently, by trying all such labellings, we can solve the recognition problem for rectilinear graphs in time $2^{{{\,\textrm{O}\,}}(n)}$. In this regard, it is worth noting that the hardness reduction from [19] can be easily modified, such that it also provides lower complexity bounds subject to the Exponential-Time Hypothesis (ETH). We shall outline this simple modification in more detail next.

The reduction from [19] transforms a ${{\,\mathrm{\textsf{3SAT}}\,}}$ instance with *n* variables and *m* clauses into a graph of size ${{\,\textrm{O}\,}}(nm)$.^6^ The main part of this graph is an L-shaped frame of size ${{\,\textrm{O}\,}}(n\,{+}\,m)$ (containing *n* connecting ports in its horizontal and *m* connecting ports in its vertical arm) and, for every variable $x_i$, a tower with *m* levels. These levels are aligned with the *m* clause-ports and are connected by edges only if the clause contains this variable or its negation. Consequently, in every variable tower for $x_i$, only those levels matter that correspond to clauses which contain $x_i$ (or $\overline{x_i}$) and the rest can be ignored. In fact, simply removing those superfluous levels result in a reduction that works in the same way, but constructs a graph of size ${{\,\textrm{O}\,}}(m)$.

With this linear reduction from ${{\,\mathrm{\textsf{3SAT}}\,}}$, it follows that the above sketched $2^{{{\,\textrm{O}\,}}(n)}$ algorithm for the recognition problem (i.e., enumerating all possible ${{\,\mathrm{\textsf{LRDU}}\,}}$-restrictions and then applying the algorithm from [28]) is optimal in the sense that the existence of a $2^{{{\,\textrm{o}\,}}(n)}$ algorithm would refute ETH.

### Combinatorial Properties of $\textsf{USGV}$

First, we shall see that the class ${{\,\mathrm{\mathrm {\textsf {USGV}}}\,}}$ is downward closed w.r.t. the subgraph relation, i.e., if $G \in {{\,\mathrm{\mathrm {\textsf {USGV}}}\,}}$, then all its subgraphs are in ${{\,\mathrm{\mathrm {\textsf {USGV}}}\,}}$. This observation will be a convenient tool for obtaining other combinatorial results.

#### Lemma 3.1

Let $G = (V, E) \in {\textsf{USGV}}$, let $v \in V$ and $e \in E$. Then $(V, E \setminus \{e\}) \in \text {\textsf{USGV}}$ and $(V \setminus \{v\}, E) \in {\textsf{USGV}}$.

#### Proof

We first prove the first statement. To this end, let $e = \{u, v\}$, where *u* and *v* are represented by unit squares $R_u$ and $R_v$ at coordinates $(x_u, y_u)$ and $(x_v, y_v)$, respectively, and, without loss of generality, we assume that $R_u {{\,\mathrm{\downarrow }\,}} R_v$ (note that this implies $x_u = x_v$). We now modify the layout as follows. Every unit square *R* on a coordinate (*x*, *y*) with $x > x_v$ or $x = x_v$ and $y \le y_v$ is moved one unit to the right (note that this means that $R_{v}$ is also moved to the right, but $R_{u}$ is not). Obviously, this modification cannot create any new visibilities and the only visibilities that are destroyed are between unit squares *R* and $R'$ on coordinates $(x_v, y)$ and $(x_v, y')$ with $y > y_v$ and $y' \le y_v$, but the only unit squares that satisfy this condition are $R_u$ and $R_v$. Consequently, the modified layout represents $(V, E \setminus \{e\})$.

In order to show the second statement, we observe that removing $R_v$ (the unit square for *v*) from the layout results in a layout for $(V \setminus \{v\}, E \cup E')$, where $E'$ is a set of at most two edges not present in $(V \setminus \{v\}, E)$. These additional edges can successively be deleted as described above, in order to obtain a layout for $(V \setminus \{v\}, E)$. $\square$

The following limitations of ${{\,\mathrm{\mathrm {\textsf {USGV}}}\,}}$ are straightforward.

#### Lemma 3.2

Let $G = (V, E) \in {\textsf{USGV}}$. Then, (i) the maximum degree of *G* is 4, (ii) for every $u, v \in V$, $|N(u) \cap N(v)| \le 2$, and (iii) for every $\{u, v\} \in E$, $N(u) \cap N(v) = \emptyset$.

#### Proof

In a grid layout, any unit square can see at most four other squares; thus, the maximum degree of *G* is 4. Let $u, v \in V$ be represented by unit squares $R_u$ and $R_v$ on coordinates $(x_u,y_u)$ and $(x_v,y_v)$, respectively. If $x_u=x_v$ or $y_u=y_v$, then there is at most one unit square that can see both $R_u$ and $R_v$. If $x_u\ne x_v$ and $y_u\ne y_v$, then there are at most two unit squares that can see both $R_u$ and $R_v$. This implies the second statement. If $R_u$ sees $R_v$, then it is impossible for any unit square to see both $R_u$ and $R_v$, which implies the third statement. $\square$

A consequence of Lemma 3.2 is that no graph from ${{\,\mathrm{\mathrm {\textsf {USGV}}}\,}}$ contains $K_{1,5}$, $K_{2,3}$, or $K_3$ as a subgraph, since they violate the first, second and third condition of Lemma 3.2, respectively. Obvious examples for graphs from ${{\,\mathrm{\mathrm {\textsf {USGV}}}\,}}$ are subgraphs of a grid; as Lemma 3.1 shows, even non-induced subgraphs of a grid. In this context, note that the problem of deciding if a given graph is such a *partial grid graph* is equivalent to deciding if it admits a unit-length VLSI layout, which, even restricted to trees, is an ${{\,\mathrm{{\textsf{N}}{\textsf{P}}}\,}}$-hard problem; see [5] for details. Yet, ${{\,\mathrm{\mathrm {\textsf {USGV}}}\,}}$ contains more, especially non-bipartite graphs, with the smallest example being $C_5$.

#### Planarity

Next, we discuss planarity issues of unit square grid visibility graphs. Before studying the relationship between ${{\,\mathrm{\mathrm {\textsf {USGV}}}\,}}$ and the class of planar graphs, we discuss the relationship between the planarity of graphs from ${{\,\mathrm{\mathrm {\textsf {USGV}}}\,}}$ and planarity of their respective layouts (where a layout is called *planar* if it does not contain any crossing visibilities). Obviously, the planarity of a layout is sufficient for the planarity of the graph it represents, while the converse does not hold (i.e., examples of non-planar layouts that nevertheless represent planar graphs can be easily found). A somewhat surprising observation in this regard is that there are also examples of planar graphs in ${{\,\mathrm{\mathrm {\textsf {USGV}}}\,}}$, for which every possible layout is necessarily non-planar (thus, existence of planar layouts is only sufficient, but not necessary for the planarity of graphs from ${{\,\mathrm{\mathrm {\textsf {USGV}}}\,}}$).

##### Proposition 3.3

**Fig. 2:**
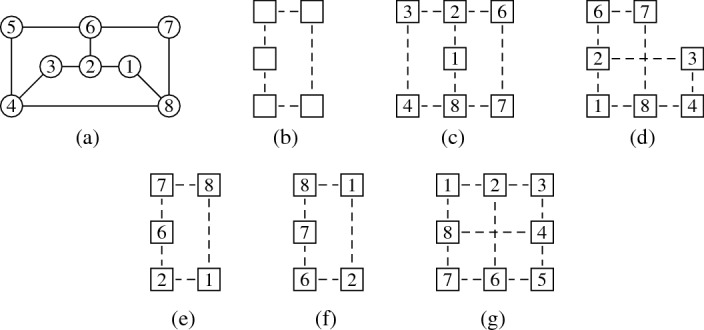
Illustrations for the proof of Proposition 3.3

Let *G* be the graph of Fig. 2(a). Then $G \in {\textsf {USGV}}$, but there exists no planar unit square grid layout for *G*.

##### Proof

The proof shall be illustrated by Fig. 2. We first consider the $C_5$ on the vertices 1, 2, 6, 7, 8 which requires a visibility layout V-isomorphic to Fig. 2(b). (c)–(g) of Fig. 2 demonstrate attempts to create a layout for *G* with all possibilities to represent the $C_5$ subgraph on vertices 1, 2, 6, 7, 8 with the layout from Fig. 2(b). Cases (c) and (d) show the only possibility to add the vertices 3 and 4 which leads to a layout where vertex 5 cannot be added with visibility to both 4 and 6. For cases (e) and (f) it is already impossible to add the vertices 3 and 4 such that they build a $C_5$ with vertices 1, 2, and 8. The only possible layout is the non-planar Fig. 2(g) which, up to V-isomorphism, is the only unit square grid representation for the graph *G*. $\square$

**Fig. 3:**
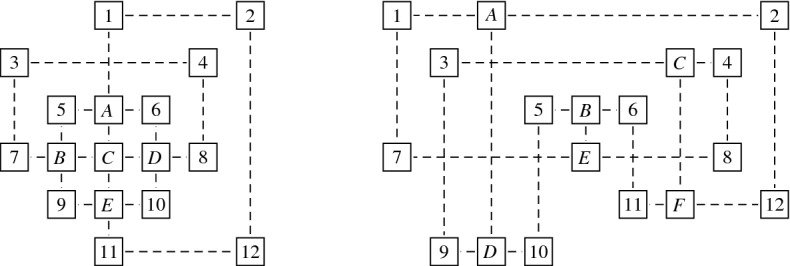
Grid layouts representing subdivisions of $K_5$ and $K_{3,3}$ (squares labeled with $A, B, \ldots$ represent the vertices of $K_5$ and $K_{3,3}$, while vertices labeled with $1, 2, \ldots$ represent subdivisions)

Regarding the relationship between ${{\,\mathrm{\mathrm {\textsf {USGV}}}\,}}$ and the class of planar graphs, we first note that, due to the degree restriction of ${{\,\mathrm{\mathrm {\textsf {USGV}}}\,}}$, there are simple planar graphs that cannot be represented by a unit square grid layout. Since the class ${{\,\mathrm{\mathrm {\textsf {USGV}}}\,}}$ is characterised in terms of drawings in two-dimensional euclidean space that are strongly restricted with respect to the crossings of their edges, it might be tempting to assume that graphs in ${{\,\mathrm{\mathrm {\textsf {USGV}}}\,}}$ are necessarily planar. However, as demonstrated by Fig. 3, ${{\,\mathrm{\mathrm {\textsf {USGV}}}\,}}$ contains a subdivision of $K_5$ and $K_{3,3}$. Hence, with Kuratowski’s theorem, we conclude the following:

##### Theorem 3.4

${{{\,\mathrm{\mathrm {\textsf {USGV}}}\,}}}$ contains non-planar graphs.

Consequently, ${{\,\mathrm{\mathrm {\textsf {USGV}}}\,}}$ and the class of planar graphs are incomparable.

We conclude this subsection by observing that unit square grid visibility graphs necessarily satisfy a slightly weaker condition of planarity, namely quasiplanarity. More precisely, a graph is *k*-quasiplanar, if it admits a drawing in which no *k* edges pairwise cross each other, and 3-quasiplanar graphs are simply called *quasiplanar*; note that 2-quasiplanar graphs coincide with planar graphs (see [26, 27]). Indeed, every unit square grid layout has at most two pairwise crossing visibilities and therefore represents a quasiplanar drawing of the graph.

#### Characterisations

Next, we investigate possibilities to characterise ${{\,\mathrm{\mathrm {\textsf {USGV}}}\,}}$. In this regard, we first observe that a characterisation by forbidden induced subgraphs is not possible (note that under the assumption ${{\,\mathrm{{\textsf{P}}}\,}}\ne {{\,\mathrm{{\textsf{N}}{\textsf{P}}}\,}}$, this also follows from the hardness of recognition).

##### Theorem 3.5

${{{\,\mathrm{\mathrm {\textsf {USGV}}}\,}}}$ does not admit a characterisation by a finite number of forbidden induced subgraphs.

##### Proof

Consider the family of graphs $\{G_n \,|\,n \ge 3\}$, where $G_n=(V_n,E_n)$ with

$$\begin{aligned} V_n&=\{u_1,\dots ,u_n\}\cup \{v_2,\dots ,v_n\}\cup \{w\}\qquad \text {and}\\ E_n&=\{\{u_i, u_{i+1}\}, \{v_i, v_{i+1}\}, \{u_i, v_i\} \mid 2 \le i \le n-1\}\\&\qquad \cup \{\{u_1, u_2\}, \{u_n, v_n\}, \{u_1, w\}, \{v_n, w\}\}. \end{aligned}$$

We note that, for every $n\ge 3$, a grid layout for $G_n-w$ (the graph created from $G_n$ by deleting the vertex *w* and its incident edges) can be constructed by placing the unit squares for the vertices $u_i$, $1\le i\le n$, on a horizontal line in this order and the unit squares for the vertices $v_i$, $2\le i\le n$, on a parallel horizontal line in this order, so that, for every *i*, $2\le i\le n$, the unit squares for $u_i$ and $v_i$ align vertically. Furthermore, every grid layout for $G_n - w$ has either this structure or places the unit squares analogously on two parallel vertical lines (i.e., it is V-isomorphic to this structure). This consideration not only shows that $G_n - w \in {{\,\mathrm{\mathrm {\textsf {USGV}}}\,}}$, but also demonstrates that $G_n \notin {{\,\mathrm{\mathrm {\textsf {USGV}}}\,}}$, since it is impossible for a unit square to see both the unit squares for $u_1$ and $v_n$. In the following, we observe that, for every $x \in V_n \setminus \{w\}$, $G_n - x \in {{\,\mathrm{\mathrm {\textsf {USGV}}}\,}}$. For $x \in \{u_1, v_2, v_n, u_n\}$, this property can be easily verified. For $x=u_i$, $2\le i\le n-1$, we can construct a grid layout by rotating the part representing vertices $\{u_1, \ldots , u_{i-1}, v_2, \ldots , v_{i-1}\}$ by ninety degrees, and an analogous construction applies in the case $x = v_i$, $3 \le i \le n-1$.

By Lemma 3.1, it follows that, for every $n \ge 3$, every proper subgraph of $G_n$ is in ${{\,\mathrm{\mathrm {\textsf {USGV}}}\,}}$, while $G_n \notin {{\,\mathrm{\mathrm {\textsf {USGV}}}\,}}$. Consequently, it is not possible to characterise ${{\,\mathrm{\mathrm {\textsf {USGV}}}\,}}$ by a finite number of forbidden induced subgraphs. $\square$

By Lemma 3.2, the classes of cycles, complete graphs and complete bipartite graphs within ${{\,\mathrm{\mathrm {\textsf {USGV}}}\,}}$ are easily characterised: $C_i \in {{\,\mathrm{\mathrm {\textsf {USGV}}}\,}}$ if and only if $i\ge 4$, $K_i\in {{\,\mathrm{\mathrm {\textsf {USGV}}}\,}}$ if and only if $i\le 2$, $K_{i,j}\in {{\,\mathrm{\mathrm {\textsf {USGV}}}\,}}$ (with $i \le j$) if and only if ($i = 1$ and $j \le 4$) or ($i = 2$ and $j = 2$). Furthermore, the trees in ${{\,\mathrm{\mathrm {\textsf {USGV}}}\,}}$ have a simple characterisation as well:

##### Theorem 3.6

A tree *T* is in $\text {\textsf{USGV}}$ if and only if the maximum degree of *T* is at most four.

##### Proof

The *only if* direction follows from Lemma 3.2. To prove the *if* direction, let $T \in {{\,\mathrm{\mathrm {\textsf {USGV}}}\,}}$ be a tree with a vertex *v* of degree at most 3. In order to append a new vertex to *v*, we can place a new unit square *R* within visibility of $R_v$, the unit square for *v*, without destroying any visibilities. Possible new visibilities between *R* and other unit squares can be removed due to Lemma 3.1. The statement of the lemma follows by induction. $\square$

By definition, ${{\,\mathrm{\mathrm {\textsf {USGV}}}\,}}\subseteq {{\,\mathrm{\mathrm {\textsf {USGV}}}\,}}_{{{\,\mathrm{{\textsf{w}}}\,}}}$ and every $G' \in {{\,\mathrm{\mathrm {\textsf {USGV}}}\,}}_{{{\,\mathrm{{\textsf{w}}}\,}}}$ can be obtained from some $G \in {{\,\mathrm{\mathrm {\textsf {USGV}}}\,}}$ by deleting some edges. Consequently, by Lemma 3.1, we conclude the following.

##### Theorem 3.7

$${{{\,\mathrm{\mathrm {\textsf {USGV}}}\,}}= {{\,\mathrm{\mathrm {\textsf {USGV}}}\,}}}_{{{\,\mathrm{{\textsf{w}}}\,}}}$$.

### Area-Minimisation Recognition Problem

The *area-minimisation* version of the recognition problem is to decide whether a given graph has a drawing or layout of given width and height. The hardness of recognition for ${{\,\mathrm{\mathrm {\textsf {USGV}}}\,}}$ and also for ${{\,\mathrm{{\textsf{H}}{\textsf{V}}}\,}}$-restricted ${{\,\mathrm{\mathrm {\textsf {USGV}}}\,}}$ carries over to the area-minimisation version, since an *n*-vertex graph has a layout if and only if it has a $(2n-1) \times (2n-1)$ layout. On the other hand, in the ${{\,\mathrm{\textsf{LRDU}}\,}}$-restricted rectilinear (or unit square grid) case, recognition can be solved in polynomial time, so the authors of [28] provide a hardness reduction that proves the area-minimisation recognition problem ${{\,\mathrm{{\textsf{N}}{\textsf{P}}}\,}}$-complete even for ${{\,\mathrm{\textsf{LRDU}}\,}}$-restricted rectilinear graphs. However, this construction does not carry over to ${{\,\mathrm{\mathrm {\textsf {USGV}}}\,}}$, since the non-edges of a rectilinear drawing translate into non-visibilities, which require space as well;^7^ moreover, it does not even work for the weak case of ${{\,\mathrm{\mathrm {\textsf {USGV}}}\,}}$, due to the necessary scaling by factor 2 to translate a rectilinear drawing into an equivalent weak unit square grid layout.

Next, we provide a reduction to show the hardness of the area-minimisation version of ${{\,\mathrm{\textsc {Rec}}\,}}({{\,\mathrm{\mathrm {\textsf {USGV}}}\,}}_{{{\,\mathrm{{\textsf{w}}}\,}}})$, which shall also imply several additional results. We first define the following problem:

3-Partition ($${{\,\mathrm{\textsf{3Part}}\,}}$$)

*Instance*: $B \in {\mathbb {N}}$ and a multi-set $A = \{a_1, a_2, \ldots , a_{3m}\} \subseteq {\mathbb {N}}$ with ${B}/{4}< a_i <{B}/{2}$, $1\le i\le 3m$, and $\sum ^{3m}_{i = 1} a_i = m B$.

*Question*: Can *A* be partitioned into multi-sets $A_1, \ldots , A_m$, such that for each *j*, $1 \le j \le m$, $\sum _{a \in A_j} a = B$?

**Fig. 4:**
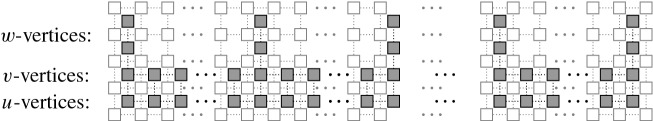
Unit square grid layout for the union of the graphs $G_f$ (solid squares) and $G_b$ (non-solid squares)

Note that the restriction ${B}/{4}< a_i <{B}/{2}$ enforces $|A_j| = 3$, $1\le j\le m$. Furthermore, by simple scaling, we can assume that $a_i > 2$, $1 \le i \le 3m$. Let $B \in {\mathbb {N}}$ and $A = \{a_1, a_2, \ldots , a_{3m}\} \subseteq {\mathbb {N}}$ be a ${{\,\mathrm{\textsf{3Part}}\,}}$ instance. We first construct a *basis graph* $G_b=(V_b,E_b)$ on $5(mB+m+2)$ vertices that form a $5\times (mB+m+2)$-grid, and a *frame graph* $G_f = (V_f, E_f)$ (see Fig. 4 for an illustration of the union of $G_b$ and $G_f$) with

$$\begin{aligned} V_f&=\{u_{i, j}, v_{i, j}, w_{i, 1}, w_{i, 2} \mid 1 \le i \le m,\,0 \le j \le B\}\\&\qquad \cup \{u_{m + 1, 0}, v_{m + 1, 0}, w_{m+1, 1}, w_{m + 1, 2}\},\\ E_f&=\{\{u_{i,j}, u_{i,j+1}\}, \{v_{i,j}, v_{i,j+1}\} \mid 1 \le i \le m, \,0 \le j \le B-1\}\\&\qquad \quad \cup \{\{u_{i, B}, u_{i+1, 0}\}, \{v_{i, B}, v_{i+1, 0}\} \mid 1 \le i \le m\}\\&\qquad \quad \cup \{\{u_{i,j}, v_{i,j}\}\mid 1 \le i \le m,\, 1 \le j \le B\}\\&\qquad \quad \cup \{\{u_{i,0}, v_{i,0}\}, \{v_{i,0}, w_{i,1}\}, \{w_{i,1}, w_{i,2}\} \mid 1 \le i \le m+1\}. \end{aligned}$$

Next, we define a graph $G_A = (V_A, E_A)$ with

$$\begin{aligned} V_A&=\bigcup ^{3m}_{i = 1} \,\{b_{i, j}, c_{i, j} \mid 1 \le j \le a_i\},\\ E_A&=\{\{b_{i, j}, b_{i, j+1}\}, \{c_{i, j}, c_{i, j+1}\} \mid 1 \le i \le 3m,\, 1 \le j \le a_i-1\}\\&\qquad \qquad \cup \{\{b_{i, j}, c_{i, j}\} \mid 1 \le i \le 3m, \,1 \le j \le a_i\}. \end{aligned}$$

Finally, we let $G = (V, E)$ with $V = V_b\cup V_f \cup V_A$ and $E = E_b\cup E_f \cup E_A$.

#### Lemma 3.8

(*B*, *A*) is a positive ${{{\,\mathrm{\textsf{3Part}}\,}}}$-instance if and only if *G* has a ${(2(mB+m)+3)} \times 9$ unit square grid layout.

#### Proof

First of all, note that there is only one possibility to represent the basis graph $G_b$ by a ${(2(mB+m)+3) }\times 9$ unit square grid layout. Considering our layout to be normalized with the lexicographically smallest index being (0, 0), this layout places a square on every *even coordinate*, i.e., (2*i*, 2*j*) with $0\le i\le mB+m+1$, $0\le j\le 4$. This directly implies that squares for the remaining vertices of *G* have to be at *odd coordinates*, i.e., $(2i+1,2j+1)$ for some $0\le i\le mB+m$, $0\le j\le 3$. For the sake of convenience, in the following, we denote the vertices $u_{i, j}$, $1 \le i \le m, 0 \le j \le B$, and $u_{m + 1, 0}$ by *u*-*vertices*, the vertices $v_{i, j}$, $1 \le i \le m$, $0 \le j \le B$, and $v_{m + 1, 0}$ by *v*-*vertices* and the vertices $w_{i, 1}, w_{i, 2}$, $1 \le i \le m + 1$, by *w*-*vertices*.

We now assume that $A_1, \ldots , A_m$ is a partition of *A* with $\sum _{a \in A_i} a= B$, $1 \le i \le m$. We can construct a $(2(mB+m)+3) \times 9$ unit square grid layout for *G* as follows. We first represent $G_b$ in the only possible way, by using all even coordinates. Then, we add squares for the vertices of $G_f$. We represent all *u*- and *v*-vertices as a horizontal “ladder”, as illustrated in Fig. 4, where vertex $u_{1,0}$ is positioned at coordinate (1, 1). All *w*-vertices can then be placed above their adjacent *v*-vertices (see Fig. 4). In the thus obtained layout, for every *i*, $1 \le i \le m + 1$, the unit squares for $u_{i, 0}$, $v_{i, 0}$, $w_{i, 1}$, $w_{i, 2}$ are positioned at $(p_i, 1)$, $(p_i, 3)$, $(p_i, 5)$, $(p_i, 7)$, respectively, where for every *i*, $1 \le i \le m + 1$, we use $p_i = (i-1) \cdot 2(B+1)+1$. Consequently, for every *i*, $1 \le i \le m$, and $\ell \in \{5,7\}$, the coordinates $(p_i + 2,\ell ), (p_i + 4,\ell ), \ldots , (p_i + 2B,\ell )$ are free (note that these are the only remaining free odd coordinates). Now let $A_i = \{a_{q_{i, 1}}, a_{q_{i, 2}}, a_{q_{i, 3}}\}$, $1 \le i \le m$. Since $a_{q_{i, 1}} + a_{q_{i, 2}} + a_{q_{i, 3}} = B$, the three connected components on vertices $b_{q_{i, r}, s}$ and $c_{q_{i, r}, s}$, $1 \le r \le 3$, $1 \le s \le a_{q_{i, r}}$, can be placed horizontally on the free coordinates $(p_i + 2,\ell ), (p_i + 4,\ell ), \ldots , (p_i + 2B,\ell )$, $\ell \in \{5,7\}$. This constructs a $(2(mB+m)+3) \times 9$ unit square grid layout for *G*.

In order to prove the other direction, we assume that there is a $(2(mB+m)+3) \times 9$ unit square grid layout for *G*. With $G_b$ fixed, the squares from $G_f$ and $G_A$ have to be placed on odd coordinates. We first note that, in any such layout, the unit squares for the *u*- and *v*-vertices must be represented as a horizontally or vertically oriented “ladder” and the same holds for the subgraphs on vertices $b_{i, j}$ and $c_{i, j}$. Moreover, since the layout has height 9, we can further assume that the orientation for the ladder of *u*- and *v*-vertices is horizontal, which also means that the orientation for the ladders of vertices $b_{i, j}$ and $c_{i, j}$ is horizontal (note that we assume that $a_i > 2$, $1 \le i \le 3m$). Due to the fact that the layout has width $2(mB+m)+3$ where the even coordinates are already blocked, all $mB+m+1$ many *u*-vertices have to be placed on coordinates $(2i+1, y_u)$, for $0 \le i \le mB+m$ and for some $y_u\in \{1,3,5,7\}$ and all $mB+m+1$ many *v*-vertices are placed on coordinates $(2i+1, y_v)$ for $0 \le i \le mB+m$ and for some $y_v\in \{1,3,5,7\}$ with $y_u\ne y_v$. Without loss of generality, we assume $y_v>y_u$.

Since, for every *i*, $1 \le i \le m + 1$, the edge $\{v_{i, 0}, w_{i, 1}\}$ must be realised by a visibility of the form $R_{w_{i,1}}{{\,\mathrm{\downarrow }\,}} R_{v_{i, 0}}$ (note that the other three visibilities of $R_{v_{i, 0}}$ are already used for all $1<i\le m$, and for $R_{v_{1,0}}$ and $R_{v_{m+1,0}}$ another horizontal visibility would exceed the width of $2(mB+m)+3$), we conclude that $y_v \le 5$. The ladders from $G_A$ require two adjacent odd *y*-coordinates which are not blocked by the *u*- and *v*-vertices. With $y_u<y_v$ and $y_v\le 5$, this is only possible if $y_u=1$ and $y_v=3$, to keep 5 and 7 as options for *y*-coordinates of the squares for the vertices in $G_A$. For every *i*, $1 \le i \le m+1$, we have $R_{w_{i, 1}} {{\,\mathrm{\downarrow }\,}} R_{v_{i, 0}}$ and either $R_{w_{i, 2}} {{\,\mathrm{\downarrow }\,}} R_{w_{i,1}}$ or $R_{w_{i,1}}{{\,\mathrm{\leftrightarrow }\,}}R_{w_{i, 2}}$. As mentioned above, for every *i*, $1 \le i\le 3m$, the subgraph on vertices $b_{i, j}, c_{i, j}$, $1\le j \le a_i$, is represented by a horizontal ladder. In total, these require exactly *mB* many squares to be placed with *y*-coordinate 5 and also *mB* many with *y*-coordinate 7. In total, there are only $mB+m+1$ odd coordinates with *y*-coordinate 5 and $m+1$ of those are already occupied by $R_{w_{i, 1}}$, $1 \le i \le m+1$. Hence we conclude that $R_{w_{i,2}}{{\,\mathrm{\downarrow }\,}} R_{w_{i, 1}}$ and thus $G_f$ and $G_b$ are represented as illustrated in Fig. 4.

Note that for *y*-coordinate 5 and 7, the *x*-coordinates $p_r = (r-1) \cdot 2(B+1)+1$ for $1\le r\le m+1$ are already occupied by the *w*-vertices. To ensure all visibilities, a ladder that represents $b_{i, j}, c_{i, j}$, $1 \le j \le a_i$, has to be placed on adjacent *x*-coordinates strictly between $p_{r}$ and $p_{r+1}$ for some $1\le r\le m+1$. Placing all vertices in $G_A$ hence requires partitioning the ladders such that exactly all *B* odd coordinates are filled between each $p_{r}$ and $p_{r+1}$. Consequently, partitioning *A* according to how the ladders are placed yields a solution for the ${{\,\mathrm{\textsf{3Part}}\,}}$-instance (*B*, *A*). $\square$

Since the reduction defined above is polynomial in *m* and *B*, and ${{\,\mathrm{\textsf{3Part}}\,}}$ is strongly ${{\,\mathrm{{\textsf{N}}{\textsf{P}}}\,}}$-complete (see [24, Thm. 4.4]), we can conclude the following:

#### Theorem 3.9

The area-minimisation variant of ${{\,\mathrm{\textsc {Rec}}\,}}({{{\,\mathrm{\mathrm {\textsf {USGV}}}\,}}}_{{{\,\mathrm{{\textsf{w}}}\,}}})$ is ${\textsf{NP}}$-complete.

The area minimisation variant implicitly solves the general recognition problem, so the question arises whether it is also hard to decide if a graph from ${{\,\mathrm{\mathrm {\textsf {USGV}}}\,}}_{{{\,\mathrm{{\textsf{w}}}\,}}}$ (given as a layout) can be represented by a layout satisfying given size bounds. Since our reduction always produces a graph that has an obvious layout as a ${{\,\mathrm{\mathrm {\textsf {USGV}}}\,}}_{{{\,\mathrm{{\textsf{w}}}\,}}}$, i.e., one that places the representation of $G_A$ independently of the frame graph, the problem remains hard even if the input graph is given as a layout.

#### Corollary 3.10

The area-minimisation variant of ${{\,\mathrm{\textsc {Rec}}\,}}({{{\,\mathrm{\mathrm {\textsf {USGV}}}\,}}}_{{{\,\mathrm{{\textsf{w}}}\,}}})$ is ${\textsf{NP}}$-complete, even if the input graph is given as a unit square grid layout.

Moreover, the problem is still ${{\,\mathrm{{\textsf{N}}{\textsf{P}}}\,}}$-complete for the ${{\,\mathrm{\textsf{LRDU}}\,}}$-restricted variant (the ${{\,\mathrm{\textsf{LRDU}}\,}}$-restriction then simply enforces the structure shown in Fig. 4).

#### Corollary 3.11

The $\textsf{LRDU}$-restricted area-minimisation variant of ${{\,\mathrm{\textsc {Rec}}\,}}({{{\,\mathrm{\mathrm {\textsf {USGV}}}\,}}}_{{{\,\mathrm{{\textsf{w}}}\,}}})$ is ${{{\,\mathrm{{\textsf{N}}{\textsf{P}}}\,}}}$-complete.

The reduction also yields a (substantially simpler) alternative proof for the hardness of the area-minimisation recognition problem for ${{\,\mathrm{\textsf{LRDU}}\,}}$-restricted rectilinear graphs [28] (more precisely, it can be shown that (*B*, *A*) is a positive ${{\,\mathrm{\textsf{3Part}}\,}}$-instance if and only if *G* has a $(2(mB+m)+3) \times 9$ rectilinear drawing), and the hardness also carries over to the variant where the input graph is already given as a rectilinear drawing.

We conclude this section by pointing out that it is open whether the ${{\,\mathrm{\textsf{LRDU}}\,}}$-restricted area-minimisation variant of ${{\,\mathrm{\textsc {Rec}}\,}}({{\,\mathrm{\mathrm {\textsf {USGV}}}\,}})$ can be solved in polynomial-time. Intuitively, reducing the size of a rectilinear drawing is difficult, since space can be saved by placing non-adjacent vertices on the same line, which is not possible for *non-weak* unit square grid layouts. However, computing a unit square grid layout of minimum size includes finding out to what extend the scaling by 2 is really necessary, which seems difficult as well.

## Unit Square Visibility Graphs

**Fig. 5:**
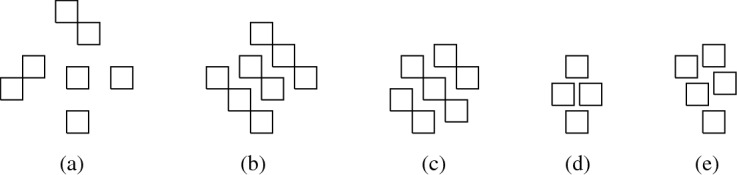
Visibility layouts for $K_{1, 6}$, $K_{2,6}$, $K_{3,4}$, $K_4$, and a $K_5$ with one missing edge

Obviously, a larger class of graphs can be represented if the unit squares are not restricted to integer coordinates (see Fig. 5 for some examples). In [12], cycles, complete graphs, complete bipartite graphs and trees in ${{\,\mathrm{\textsf{USV}}\,}}$ are characterised as follows:

- $$C_i \in {{\,\mathrm{\textsf{USV}}\,}}$$, for every $$i \in {\mathbb {N}}$$,
- $K_i \in {{\,\mathrm{\textsf{USV}}\,}}$ if and only if $i \le 4$,
- $K_{i, j} \in {{\,\mathrm{\textsf{USV}}\,}}$ with $i \le j$ if and only if ($1\le i\le 2$ and $i\le j\le 6$) or ($i = 3$ and $3\le j\le 4$),^8^
- a tree *T* is in ${{\,\mathrm{\textsf{USV}}\,}}$ if and only if it is the union of two subdivided caterpillar forests with maximum degree 3 (note that [25] provides an algorithm that efficiently checks this property).

Next, we observe that every graph with at most four vertices is in ${{\,\mathrm{\textsf{USV}}\,}}$, while $K_5$ is not.

### Proposition 4.1

Every graph with at most four vertices is in ${{{\,\mathrm{\textsf{USV}}\,}}}$.

### Proof

**Fig. 6:**
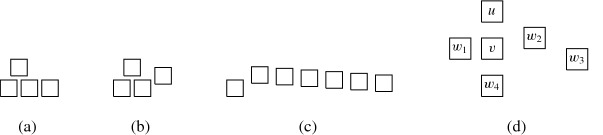
Some visibility layouts

It is straightforward to construct layouts for graphs with at most three vertices (thus, also for graphs with four vertices that are not connected) and for $P_4$, $C_4$, and $K_{1, 3}$. This only leaves $K_4$, for which a layout is presented in Fig. 5, and the two graphs represented by the layouts in Fig. 6, (a) and (b). $\square$

A crucial difference between ${{\,\mathrm{\mathrm {\textsf {USGV}}}\,}}$ and ${{\,\mathrm{\textsf{USV}}\,}}$ is that for the latter, the degree is not bounded, as witnessed by layouts of the form shown in Fig. 6(c). However, if a unit square sees at least seven other unit squares, then these must be placed in such a way that visibilities or “paths” between some of them are enforced (note that any $K_{1,n}$ may exist as induced subgraph, as can be demonstrated by modifying the above example layout so that between each two consecutive neighbours another “visibility-blocking” unit square is inserted). In [12], it is formally proven that in graphs from ${{\,\mathrm{\textsf{USV}}\,}}$ any vertex of degree at least 7 must lie on a cycle. In particular, these observations point out that an analogue of Lemma 3.1 is not possible for ${{\,\mathrm{\textsf{USV}}\,}}$.

For the class of trees within ${{\,\mathrm{\textsf{USV}}\,}}$, as long as we consider trees with maximum degree strictly less or larger than 6, a much simpler characterisation (compared to the one mentioned at the beginning of this section) applies:

### Theorem 4.2

Let *T* be a tree with maximum degree *k*. If $k \le 5$, then $T \in {{{\,\mathrm{\textsf{USV}}\,}}}$, and if $k \ge 7$, then $T \notin {{{\,\mathrm{\textsf{USV}}\,}}}$.

### Proof

The second statement follows from the fact that for unit square visibility graphs, any vertex of degree at least‘ 7 lies on a cycle, which has been shown in [12]. Let $T\in {{\,\mathrm{\textsf{USV}}\,}}$ be a tree with a maximum degree of 5 represented by a layout ${\mathcal {R}}$. We show that if we append at most four nodes to an arbitrary leaf of *T*, the resulting tree can still be represented by a layout. The first statement of the lemma follows then by induction. Let *v* be a leaf of *T* with a parent node *u* and let $R_v, R_u \in {\mathcal {R}}$ be the corresponding unit squares. Without loss of generality, we assume that $R_u{{\,\mathrm{\downarrow }\,}} R_v$. Next, we note that that there is no $R \in {\mathcal {R}}$ with $R {{\,\mathrm{\rightarrow }\,}}R_v$, $R_v {{\,\mathrm{\rightarrow }\,}}R$, or $R_v{{\,\mathrm{\downarrow }\,}} R$, which, in particular, means that $R_{v}$ can be moved arbitrarily far down without destroying or introducing any visibilities. Consequently, we can assume that the two rectangles of height 0.5 and infinite width just above and below $R_{v}$ are not intersected by any $R\in {\mathcal {R}}$. This implies that we can append new vertices $w_i$, $1 \le i \le 4$, to *v* by placing new unit squares $R_{w_i}$, $1 \le i \le 4$, as shown in Fig. 6(d). Moreover, the only new edges are between the $w_i$, $1\le i\le 4$, and *v*, and no existing edges are destroyed. Consequently, the obtained layout represents the tree $T'$ that is obtained from *T* by appending four new nodes to the leaf *v*. In a similar way, we can also append less than four new vertices to *v*. $\square$

**Fig. 7:**
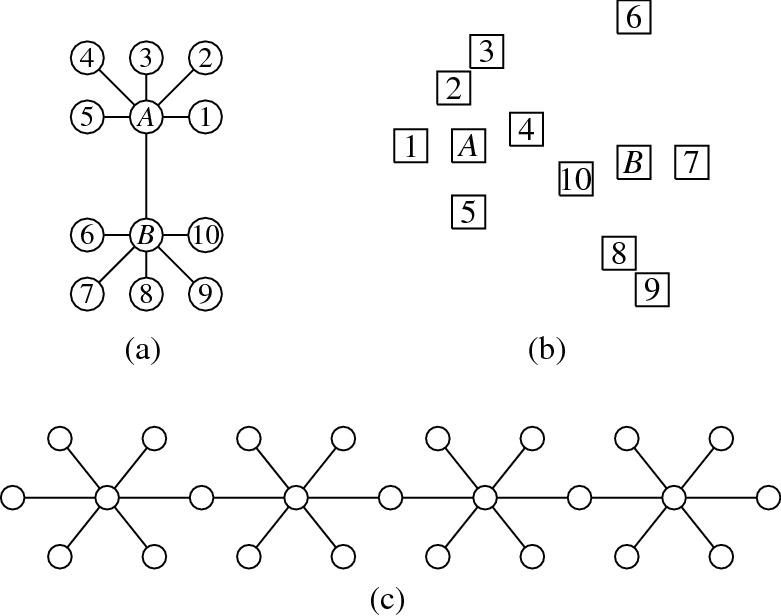
Illustration for trees from ${{\,\mathrm{\textsf{USV}}\,}}$ with maximum degree 6

That layouts for trees are rather involved as soon as there are degree-6 nodes, is pointed out by Fig. 7(a), which shows an example of a tree from ${{\,\mathrm{\textsf{USV}}\,}}$ with maximum degree 6, and its representing layout, shown in Fig. 7(b). This is due to the fact that, as can be easily verified, any node of degree 6 must be represented V-isomorphically to Fig. 5(a) (note that this also holds for nodes *A* and *B* in (a) and (b) of Fig. 7). Figure 5(a) also demonstrates that not all trees with maximum degree 6 can be represented: let *R* denote the square below the central square in the layout, then it is impossible for *R* to see five additional unit squares that exclusively see *R*. On the other hand, ${{\,\mathrm{\textsf{USV}}\,}}$ contains trees with arbitrarily many degree-6 vertices, e.g., trees of the form depicted in Fig. 7(c) (it is straightforward to see that they can be represented as the union of two forests of caterpillars with maximum degree 3). This reasoning shows that not all planar graphs are in ${{\,\mathrm{\textsf{USV}}\,}}$, while it follows from [33] that all planar graphs are (non-unit square) rectangle visibility graphs (also see [32]).^9^ Finally, we note that, unlike for the *grid* case, ${{\,\mathrm{\textsf{USV}}\,}}$ is a proper subset of ${{\,\mathrm{\textsf{USV}}\,}}_{{{\,\mathrm{{\textsf{w}}}\,}}}$ (e.g., $K_{1, 7}$ is a separating example):

### Theorem 4.3

$${{{\,\mathrm{\textsf{USV}}\,}}} \subsetneq {{{\,\mathrm{\textsf{USV}}\,}}}_{{{\,\mathrm{{\textsf{w}}}\,}}}$$.

### The Recognition Problem

The recognition problem for ${{\,\mathrm{\textsf{USV}}\,}}$ consists in checking whether a given graph can be represented by a unit square layout. We first observe that this problem is in ${{\,\mathrm{{\textsf{N}}{\textsf{P}}}\,}}$ (note that this is not completely trivial, since we cannot naively guess a layout) and the main result of this section shall be its hardness (see Theorem 4.13).

#### Theorem 4.4

$${{\,\mathrm{\textsc {Rec}}\,}}({{{\,\mathrm{\textsf{USV}}\,}}}) \in {{{\,\mathrm{{\textsf{N}}{\textsf{P}}}\,}}}$$.

#### Proof

Assuming there exists a ${{\,\mathrm{\textsf{USV}}\,}}$ layout for a graph *G* over *n* vertices, this layout can obviously be considered to use space reasonably, hence with *x*- and *y*-coordinates within range 0 to *n*. Further, squares do not have to be shifted arbitrarily: Shifting the *x*-coordinate of a rectangle *R* with respect to the *x*-coordinate of another rectangle $R'$ by more than zero but less than one is only necessary if *R* needs to see another rectangle to the same side as $R'$. The number of different shifts of distance strictly between zero and one which are necessary for a layout is hence bounded by the maximum degree of the input graph. In general, this means that if $G\in {{\,\mathrm{\textsf{USV}}\,}}$, guessing all possibilities to choose coordinates (*x*, *y*) with $x,y\in \{a/n\mid 0\le a\le n^2\}$ for each vertex in *G* yields at least one layout for *G*. Since checking if a set of coordinates yields a feasible layout for a graph *G* can be done in polynomial time, this kind of guessing *n* coordinates from a set of $(n+1)^4$ possibilities yields ${{\,\mathrm{{\textsf{N}}{\textsf{P}}}\,}}$-membership for ${{\,\mathrm{\textsc {Rec}}\,}}({{\,\mathrm{\textsf{USV}}\,}})$. For ${{\,\mathrm{\textsc {Rec}}\,}}({{\,\mathrm{\mathrm {\textsf {USGV}}}\,}})$, the similar arguments apply and it is even sufficient to only guess integer coordinates (*x*, *y*) with $0\le x,y\le 2n-1$. $\square$

The ${{\,\mathrm{{\textsf{N}}{\textsf{P}}}\,}}$-hardness proof of ${{\,\mathrm{\textsc {Rec}}\,}}({{\,\mathrm{\textsf{USV}}\,}})$ is rather involved on a technical level and we shall break it up into several parts. In the next subsection, we prove a crucial technical lemma and we explain the main parts of our reduction in an intuitive way.

#### Preliminaries for the Hardness Proof

**Fig. 8:**
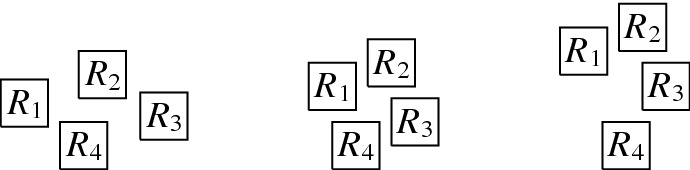
The three ways of representing $K_4$ by a layout

The complete graph $K_4$ shall be a basic building block for our reduction. Thus, we first show that, intuitively speaking, the $K_4$ is a structure that does not give too much leeway with respect to how a layout can represent it. More precisely, we show that every layout for $K_4$ is V-isomorphic to one of the three layouts of Fig. 8. Since these three possibilities are uniquely determined by the horizontal and vertical visibilities (up to a renaming of the unit squares), e.g., for the first layout of Fig. 8, we have $R_1 {{\,\mathrm{\rightarrow }\,}}\{R_2, R_3, R_4\}$, $R_2 {{\,\mathrm{\rightarrow }\,}}R_3$, $R_2{{\,\mathrm{\downarrow }\,}} R_4$, $R_4 {{\,\mathrm{\rightarrow }\,}}R_3$, we can state the lemma in the following way (note that the three cases of the following lemma correspond to the three layouts of Fig. 8).

##### Lemma 4.5

Every layout for $K_4$ is V-isomorphic to a layout $\{R_1, R_2, R_3, R_4\}$ that satisfies one of the following cases:

1. $$R_1 {{\,\mathrm{\rightarrow }\,}}\{R_2, R_3, R_4\}$$, $$R_2 {{\,\mathrm{\rightarrow }\,}}R_3$$, $$R_2 {{\,\mathrm{\downarrow }\,}} R_4$$, $$R_4 {{\,\mathrm{\rightarrow }\,}}R_3$$,
2. $$R_1 {{\,\mathrm{\rightarrow }\,}}\{R_2, R_3\}$$, $$R_1{{\,\mathrm{\downarrow }\,}} R_4$$, $$R_2{{\,\mathrm{\downarrow }\,}}\{R_3, R_4\}$$, $$R_4 {{\,\mathrm{\rightarrow }\,}}R_3$$,
3. $$R_1 {{\,\mathrm{\rightarrow }\,}}\{R_2,R_3\}$$, $$R_1{{\,\mathrm{\downarrow }\,}} R_4$$, $$R_2{{\,\mathrm{\downarrow }\,}}\{R_3,R_4\}$$, $$R_3{{\,\mathrm{\downarrow }\,}} R_4$$.

##### Proof

It can be easily verified that at least one of the edges of $K_4$ must be represented by a visibility of length strictly less than 1. Hence, we assume that this is true for the visibility between $R_1$ and $R_2$ and, furthermore, we assume that $R_1{{\,\mathrm{\rightarrow }\,}}R_2$ and that for the *y*-components $y_1$ and $y_2$ of the coordinates of $R_1$ and $R_2$, respectively, we have $y_2\le y_1$ (i.e., $R_1$ is to the left of $R_2$ and $R_2$ is either horizontally aligned with $R_1$ or further down. We now investigate all possibilities of how the remaining unit squares $R_3$ and $R_4$ can be placed in the layout in order to represent $K_4$.

- $\{R_3, R_4\}{{\,\mathrm{\updownarrow }\,}}\{R_1, R_2\}$: This implies that $R_3$ must be placed above and $R_4$ below $R_1$ and $R_2$, or vice versa:
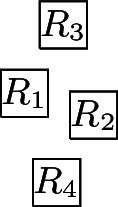
 This layout is V-isomorphic to case 1.
- $\{R_3, R_4\}{{\,\mathrm{\leftrightarrow }\,}}\{R_1, R_2\}$: If $R_3$ and $R_4$ are placed on opposite sides of $R_1$ and $R_2$, then they either cannot see each other or one of them cannot see $R_1$ or $R_2$. If they are placed on the same side of $R_1$ and $R_2$, then at most one of them can see both $R_1$ and $R_2$. Thus, this case is not possible.
- $R_3{{\,\mathrm{\leftrightarrow }\,}}\{R_1, R_2\}$ and $R_4{{\,\mathrm{\updownarrow }\,}}\{R_1, R_2\}$ or $R_3{{\,\mathrm{\updownarrow }\,}}\{R_1, R_2\}$ and $R_4{{\,\mathrm{\leftrightarrow }\,}}\{R_1, R_2\}$: We only consider the case $R_3{{\,\mathrm{\leftrightarrow }\,}}\{R_1, R_2\}$ and $R_4{{\,\mathrm{\updownarrow }\,}}\{R_1, R_2\}$, since the other case is symmetric. Since $R_1$ and $R_2$ are at horizontal distance less than 1, it follows that either $R_3 {{\,\mathrm{\rightarrow }\,}}\{R_1, R_2\}$ or $\{R_1, R_2\} {{\,\mathrm{\rightarrow }\,}}R_3$. If $R_3 {{\,\mathrm{\rightarrow }\,}}\{R_1, R_2\}$, then $\{R_1, R_2\}{{\,\mathrm{\downarrow }\,}} R_4$ and $R_3{{\,\mathrm{\rightarrow }\,}}R_4$. Analogously, $\{R_1, R_2\} {{\,\mathrm{\rightarrow }\,}}R_3$ implies that $R_4 {{\,\mathrm{\downarrow }\,}}\{R_1, R_2\}$ and $R_4{{\,\mathrm{\rightarrow }\,}}R_3$:
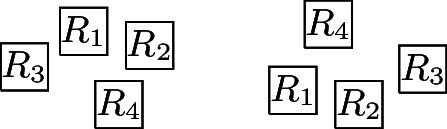
 Both these layouts are V-isomorphic to case 3.

Hence, from now on, we can assume that at least one of $R_3$ and $R_4$ is placed so that it sees one of $R_1$ and $R_2$ horizontally and the other one vertically. Without loss of generality, we assume that this is the case for $R_3$, which means that either $R_3 {{\,\mathrm{\leftrightarrow }\,}}R_1$ and $R_3{{\,\mathrm{\updownarrow }\,}} R_2$ or $R_3{{\,\mathrm{\updownarrow }\,}} R_1$ and $R_3{{\,\mathrm{\leftrightarrow }\,}}R_2$. Moreover, due to the relative positions of $R_1$ and $R_2$, this is only possible if $R_1 {{\,\mathrm{\rightarrow }\,}}R_3$ and $R_3{{\,\mathrm{\downarrow }\,}} R_2$ or $R_1{{\,\mathrm{\downarrow }\,}} R_3$ and $R_3 {{\,\mathrm{\rightarrow }\,}}R_2$. We assume the former situation and now check all possibilities of how $R_4$ can be placed in the layout in order to represent $K_4$.

- $R_4{{\,\mathrm{\updownarrow }\,}}\{R_1, R_2\}$: Since $R_1 {{\,\mathrm{\rightarrow }\,}}R_2$ (i.e., $R_4$ does not vertically fit between $R_1$ and $R_2$), either $\{R_1,R_2\}{{\,\mathrm{\downarrow }\,}} R_4$ or $R_4 {{\,\mathrm{\downarrow }\,}}\{R_1,R_2\}$. The case $\{R_1,R_2\}{{\,\mathrm{\downarrow }\,}} R_4$ implies $R_3{{\,\mathrm{\downarrow }\,}} R_4$ and thus, we have case 3. $R_4{{\,\mathrm{\downarrow }\,}}\{R_1,R_2\}$ requires that for the *x*-coordinates $x_i$ of $R_i$ we have $x_1<x_4<x_2<x_3$ and hence either $R_4{{\,\mathrm{\downarrow }\,}} R_3$ which also yields case 3., or $R_4{{\,\mathrm{\rightarrow }\,}}R_3$ which yields case 2. See also the illustrations below:
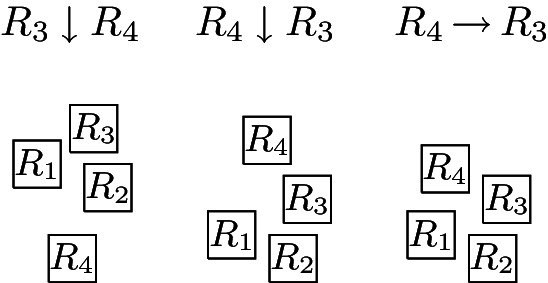
- $R_4{{\,\mathrm{\leftrightarrow }\,}}\{R_1, R_2\}$: Since $R_4$ must see $R_3$, this implies $R_1 {{\,\mathrm{\rightarrow }\,}}R_4$ and $R_2 {{\,\mathrm{\rightarrow }\,}}R_4$ which means that either $R_3{{\,\mathrm{\rightarrow }\,}}R_4$ or $R_3{{\,\mathrm{\downarrow }\,}} R_4$:
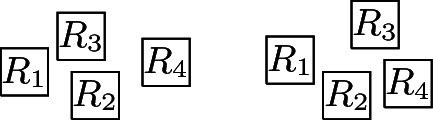
 Observe that the layout with $R_3{{\,\mathrm{\rightarrow }\,}}R_4$ is V-isomorphic to case 1., and the layout with $R_3{{\,\mathrm{\downarrow }\,}} R_4$ V-isomorphic to case 3.
- $R_4{{\,\mathrm{\leftrightarrow }\,}}R_1$, $R_4{{\,\mathrm{\updownarrow }\,}} R_2$: We note that if $R_4{{\,\mathrm{\rightarrow }\,}}R_1$, then $R_4$ cannot see $R_2$ vertically, which implies $R_1{{\,\mathrm{\rightarrow }\,}}R_4$. In particular, this also implies $R_4{{\,\mathrm{\downarrow }\,}} R_2$. This gives a layout of the following form (where $R_3$ and $R_4$ can also switch places)
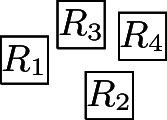
 This layout is V-isomorphic to case 3.
- $R_4{{\,\mathrm{\leftrightarrow }\,}}R_2$, $R_4{{\,\mathrm{\updownarrow }\,}} R_1$: Similarly to the previous case, if $R_4{{\,\mathrm{\downarrow }\,}} R_1$, then $R_4$ cannot see $R_2$ horizontally; thus, $R_1{{\,\mathrm{\downarrow }\,}} R_4$, which, in particular, implies $R_4 {{\,\mathrm{\rightarrow }\,}}R_2$:
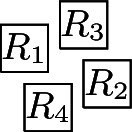
 This layout is V-isomorphic to case 2.

The case where $R_1{{\,\mathrm{\downarrow }\,}} R_3$ and $R_3 {{\,\mathrm{\rightarrow }\,}}R_2$ is symmetric to the case $R_1 {{\,\mathrm{\rightarrow }\,}}R_3$ and $R_3{{\,\mathrm{\downarrow }\,}} R_2$ considered above. Furthermore, the cases that $y_1 \le y_2$ or that the visibility between $R_1$ and $R_2$ is vertical can be handled analogously. This completes the proof. $\square$

Next, we describe the gadgets used in our reduction in an intuitive way:

**Fig. 9:**
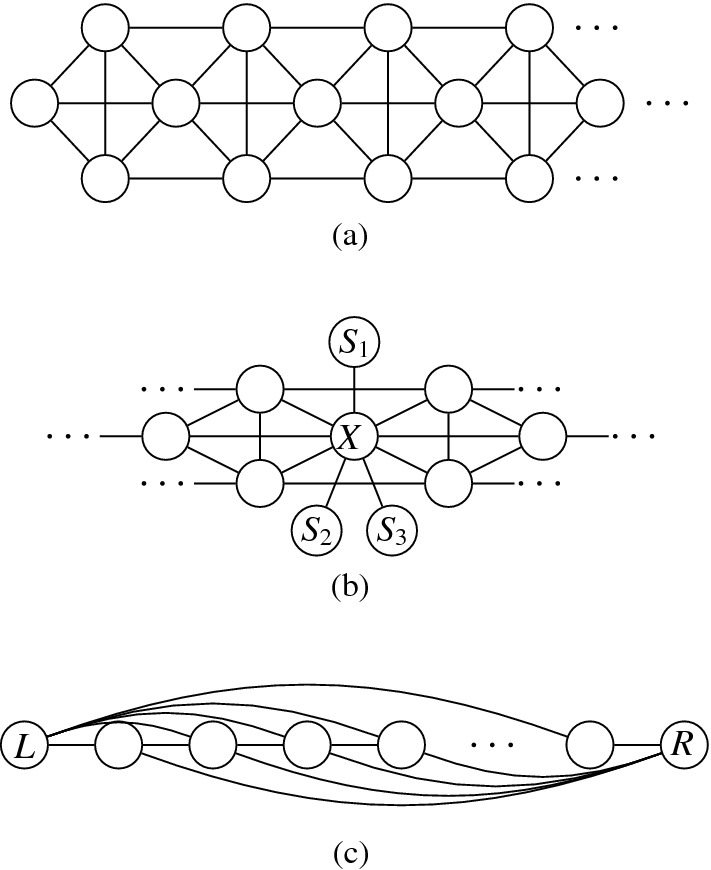
Illustration of the main gagdets (as combinatorial graphs)

**Fig. 10:**
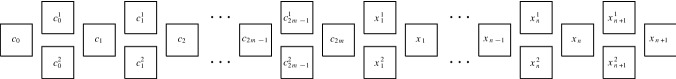
The backbone-gadget

- **Backbone gadget** As the central structure, we use a sequence of $K_4$’s as depicted in Fig. 9(a). Note that the $K_4$’s are joined in the sense that the last vertex of the $i^{\text {th}}$ $K_4$ is also the first vertex of the $(i+1)^{\text {th}}$ $K_4$, and also the upper and lower vertex of every $K_4$ is connected to the corresponding vertices of the preceding and the following $K_4$. In this structure, the *inner vertices* on the middle line (i.e., the vertices that belong to two $K_4$’s) shall be the ones that actually carry information in the reduction (see the explanation of the *selection gadget* below), while the others are merely necessary to enforce certain structural properties. An obvious visibility layout for the backbone can be obtained by just replacing the vertices in Fig. 9(a) by unit squares (see Fig. 10) and in our reduction, we will force the backbone to be represented in this way. However, since the layout of Fig. 10 is not at all the only possible one for representing the backbone, our line of reasoning will not be so simple and has to take other structural properties into account as well.
- **Selection gadget** Figure 9(b) shows two $K_4$’s of the backbone with common inner vertex *X*, which is connected to three vertices $S_1$, $S_2$, and $S_3$ (called *selectables* in the following) that are not part of the backbone. If the backbone is represented by unit squares strictly horizontally (more precisely, as shown in Fig. 10), then the visibilities of the unit squares for $S_1$, $S_2$, and $S_3$ and the unit square for *X* must be vertical (if the unit square for some $S_i$ would see *X* horizontally, then it would get in the way of the backbone; thus, causing forbidden visibilities). Moreover, if all three unit squares for $S_1$, $S_2$, and $S_3$ are on the same side of the backbone, then there would be forbidden visibilities between them, which implies that exactly one of these unit squares is above the backbone and the other two are below (or the other way around). Consequently, this implements a gadget that selects one element out of three.
- **Path gadget** Figure 9(c) shows a path from vertex *L* to vertex *R* with the special property that both *L* and *R* are connected to all internal vertices of this path. In our reduction, we shall use such paths where the internal vertices are selectables of selection gadgets. As for the backbone, we show that in the layout, such paths must expand along one dimension, which implies that the path either lies completely above or completely below the backbone; thus, implementing a kind of synchronisation between the selections done by the selection gadgets. Unfortunately, as it was the case for the backbone, the combinatorial structure of the path shown in Fig. 9(c) is not sufficient to force its layout into a strictly horizontal or vertical shape; our argument will again be non-local and dependent on other parts of the represented graph.

Having described the basic gadgets that we will use, we can now sketch the reduction. In this way, we hope to equip the reader with an understanding of the general idea of the reduction, so that the very involved technical details that follow will be easier to grasp.

We represent a monotone Boolean formula in 3-CNF by a graph as follows. We use a backbone with a first part in which the inner vertices represent the clauses and a second part in which the inner vertices represent the variables; i.e., each clause and each variable is represented by a selection gadget as described above (called *clause* and *variable gadgets* in the following). The three selectables of a clause gadget correspond to the three literals of the clause; placing a selectable above the backbone corresponds to assigning its variable the value *true* and placing it below the backbone corresponds to assigning its variable the value *false*. The situation is a bit more complicated with respect to the variable gadgets. Obviously, we want to assign either *true* or *false* to the variable, but our selection gadgets only work with three instead of two selectables. We handle this difficulty by interpreting two selectables to correspond to *false* and one to *true*. In this way, there is always at least one *false* selectable on the opposite side (with respect to the backbone) of the *true* selectable. All selectables that correspond to an occurrence of a variable $x_i$ in the formula are connected by a path gadget, as described above, that leads from the leftmost such occurrence to the *true* $x_i$-variable gadget selectable.

The correctness of the reduction can now be easily seen. The path gadget, called a variable path, for all occurrences of $x_i$ is always on one side of the backbone. Arbitrarily interpreting “above the backbone” as Boolean value *true*, this describes a valid assignment to the variables. Furthermore, for every clause, exactly one selectable is on one side of the backbone, while the other two are on the other side; thus, the corresponding assignment is a not-all-equal assignment for the input formula.

In this reduction sketch, we have assumed that the backbone stretches horizontally from left to right (or vertically, which is analogous), the variable paths are also represented horizontally and either lie completely above or completely below the backbone, exactly one of the three selectables is on one side of the backbone, while the other two are on the opposite side. As it turns out, formally proving these properties is surprisingly non-trivial and requires substantial technical effort. Before we move on to this task, let us give some intuition of the challenges that lie ahead.

The main difficulty is that proving that any layout is necessarily V-isomorphic to the one sketched above cannot be done separately for the individual gadgets, e.g., showing that the backbone must be represented as in Fig. 10 (as already mentioned, the structure of the backbone alone simply does not enforce such a layout) and the selectables must form horizontal paths and so on. Instead, the desired structure of the layout is only enforced by a rather complicated interplay of the different parts of the graph. A main building stone is our Lemma 4.5, which says that a $K_4$ can only be represented in three different ways (up to V-isomorphism). This observation is important, since the backbone is a sequence of $K_4$.

There is another technical difficulty that we have neglected in the sketches above. When reasoning about layouts for combinatorial graphs, it is tempting to exclude certain forms of layouts by demonstrating that they would necessarily place a unit square *X* “within visibility” of a unit square *Y* that represents a non-adjacent vertex. However, the visibility in a layout between unit squares *X* and *Y* does not only depend on their placement but also on the placement of other squares that may block their potential visibility. Hence, the argument from above is only correct if “placing within visibility” means that in the layout, there will necessarily be a visibility between *X* and *Y*, which is only the case if the visibility is not “blocked” by other unit squares. Consequently, this argument only works, if all other vertices that are adjacent to the ones corresponding to *X* and *Y* are taken into consideration as well. In other words, a layout that places unit squares within mutual visibility for non-adjacent vertices does not necessarily lead to a contradiction, since the forbidden visibility might be blocked by other unit squares. This difficulty further substantially increases the combinatorial depth of the already technical arguments.

#### The Reduction

In this subsection, we formally define our reduction. As already mentioned, we use the following variant of the 3-satisfiability problem (shown to be NP-hard in [30] under the name NP2 Not-All-Equal Satisfiability):

Monotone Not-All-Equal 3-Satisfiability (${{\,\mathrm{\mathsf {NAE-3SAT}}\,}}$)

*Instance*: A Boolean formula *F* in 3-CNF and without negated variables.

*Question*: Is there an assignment for the variables of *F*, such that every clause contains at least one true and one false variable?

Let $F = \{c_1, \ldots , c_m\}$ be a 3-CNF formula over the variables $x_1,\dots ,x_n$. We assume that each clause has exactly three variables and that no variable occurs more than once in any clause. We further assume that every variable occurs at least three times in the formula. Observe that every instance of Monotone Not-All-Equal 3-Satisfiability can be checked in polynomial time to ensure these properties. For the sake of convenience, let $c_i = \{y_{i, 1}, y_{i, 2}, y_{i, 3}\}$, $1 \le i \le m$.

We transform *F* into a graph $G=(V,E)$ as follows. The set of vertices is defined by $V = V_c \cup V_x\cup V_h$, where

$$\begin{aligned} V_c&=\bigl \{c_j, c^1_j,c^2_j \mid 0 \le j \le m-1\bigr \} \cup \{c_m\} \cup \bigl \{l_j^1,l_j^2,l_j^3 \mid 1 \le j \le m\bigr \},\\ V_x&=\bigl \{x_i, x^1_i, x^2_i \mid 1 \le i \le n + 1\bigr \} \cup \bigl \{t_i, \overset{_{\rightarrow }}{t_i}, \overset{_{\leftarrow }}{t_i}, f^1_i, \overset{_{\rightarrow }}{f_i^1}, \overset{_{\leftarrow }}{f_i^1}, f^2_i, \overset{_{\rightarrow }}{f_i^2}, \overset{_{\leftarrow }}{f_i^2} \mid 1 \le i \le n\bigr \},\\ V_h&=\bigl \{h_{t_i}^r,h_{f_i^1}^r,h_{f_i^2}^r \mid 1 \le i \le n, 0 \le r \le 4\bigr \}. \end{aligned}$$

The vertices $c_j,c^1_j,c^2_j$ and $x_i,x^1_i,x^2_i$ are part of the clause and variable gadgets, respectively, of the backbone, where the vertices $c_j$ and $x_i$ are the inner vertices (see Fig. 10). More formally, we require, for every $0 \le j \le m-1$ and $1 \le i \le n$, the following groups of four vertices to form a $K_4$: $\{c_j, c^1_j, c^2_j, c_{j + 1}\}$, $\{x_i, x^1_{i + 1}, x^2_{i + 1}, x_{i + 1}\}$, and $\{c_{m}, x^1_1, x^2_1, x_1\}$. Moreover, for every $j \in \{1, 2\}$, the vertices $c^j_0, c^j_1, \ldots , c^j_{m - 1}, x^j_1, x^j_2, \ldots , x^j_{n + 1}$ form a path in this order (observe that this creates all the edges of the backbone structure, as illustrated in Fig. 9(a)).

**Fig. 11:**
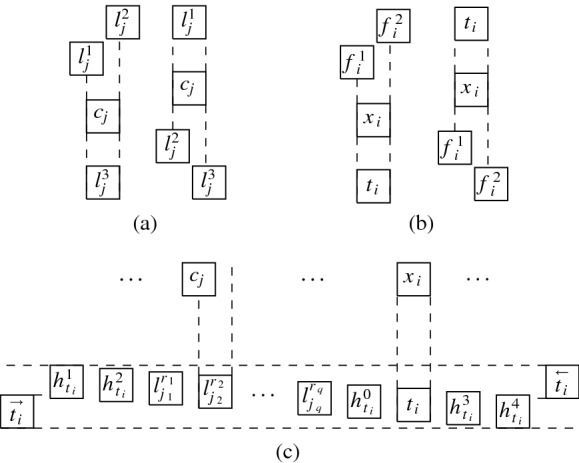
Possible placements of selectables, possible placements of assignment vertices, and the clause path for $x_i$

Vertex $t_i$ is the *true* selectable, while the $f^1_{i}$ and $f^1_{i}$ are the first and second *false* selectable of the variable gadgets for variable $x_i$. Vertices $l^1_{j}, l^2_{j}, l^3_{j}$ are the selectables of the clause gadget for clause $c_j$. Formally this means that we introduce the edges $\{x_i, t_i\}$, $\{x_i, f^1_i\}$, $\{x_i, f^2_i\}$ for all $1 \le i \le n$ and $\{c_j, l^r_j\}$ for all $1 \le j \le m$ and $1 \le r \le 3$ (note that this corresponds to the definition of the selection gadgets given in Sect. 4.1.1; see also Fig. 11, (a) and (b)). The variable paths described in Sect. 4.1.1 are obtained as follows. For every $1 \le j \le m$, $1 \le i \le n$, and $1 \le r \le 3$:

- if $y_{j, r} = x_i$, there are edges $\{l^r_j, \overset{_{\rightarrow }}{t_i}\}$, $\{l^r_j, \overset{_{\leftarrow }}{t_i}\}$,
- there are edges $\{t_i, \overset{_{\rightarrow }}{t_i}\}$, $\{t_i, \overset{_{\leftarrow }}{t_i}\}$ and $\{\overset{_{\rightarrow }}{t_i},h_{t_i}^p\}$,$\{\overset{_{\leftarrow }}{t_i},h_{t_i}^p\}$ for all $0\le p\le 4$,
- there are edges $\{f_i^s, \overset{_{\rightarrow }}{f_i^s}\}$, $\{f_i, \overset{_{\leftarrow }}{f_i^s}\}$ and $\{\overset{_{\rightarrow }}{f_i^s},h_{f_i^s}^p\}$, $\{\overset{_{\leftarrow }}{f_i^s},h_{f_i^s}^p\}$ for all $0\le p\le 4$, $s\in \{1,2\}$,

Moreover, for every *i*, $1 \le i \le n$,

- if $N(\overset{_{\rightarrow }}{t_i}) = \{h_{t_i}^1,h_{t_i}^2,l^{r_1}_{j_1}, l^{r_2}_{j_2}, \ldots , l^{r_{q}}_{j_{q}},h_{t_i}^0,t_i,h_{t_i}^3,h_{t_i}^4\}$ with $j_1< j_2< \ldots < j_q$, then these vertices form a path in this order,
- For $s\in \{1,2\}$, the vertices in $\{h_{f_i^s}^1,h_{f_i^s}^2,h_{f_i^s}^0,f_i^s,h_{f_i^s}^3,h_{f_i^s}^4\}$ form a path in this order.

We note that this constructs path gadgets as defined in Sect. 4.1.1; see also Fig. 11(c). This concludes the definition of the reduction, a full example can be found in Sect. A.2.

#### Proof of Correctness

It remains to prove the correctness of the reduction, i.e., the CNF formula *F* has a not-all-equal assignment if and only if $G \in {{\,\mathrm{\textsf{USV}}\,}}$. We start with the *only if* direction, which is the easier one.

We assume that the formula *F* is not-all-equal satisfiable and show how a layout for *G* can be constructed. First, we represent the backbone as illustrated in Fig. 10. If a variable $x_i$ is assigned the value *true*, then we place the unit squares $R_{\{x_i, t_i, f^1_{i}, f^2_{i}\}}$ as illustrated on the left side of Fig. 11(b), and otherwise as illustrated on the right side. The edges for the vertices $t_i, \overset{_{\rightarrow }}{t_i}, \overset{_{\leftarrow }}{t_i}, h_{t_i}^r$, $0 \le r \le 4$, and all $l^r_{j}$ with $y_{j, r} = x_i$ can be realised as illustrated in Fig. 11(c) (either placed above or below the backbone, according to the position of $R_{t_i}$). The paths must be horizontally shifted so that they can see their corresponding $R_{c_j}$ from above or from below, according to whether the path lies above or below the backbone (as indicated in Fig. 11(c)). As long as not all paths for the three variables of the same clause lie all above or all below the backbone, this is possible by arranging the unit squares as illustrated in Fig. 11(a). However, if for some clause all paths lie on the same side of the backbone, then the variables of the clause are either all set to *true* or all set to *false*, which is a contradiction to the assumption that the assignment is not-all-equal satisfiable. Consequently, we can represent *G* as described, which yields the following lemma. A formal definition of the layout is provided in Sect. A.1.

##### Lemma 4.6

If *F* is not-all-equal satisfiable, then $G \in {{{\,\mathrm{\textsf{USV}}\,}}}$.

Proving that a layout for *G* translates into a satisfying not-all-equal assignment for *F*, is much more involved (see the discussions and sketches given in Sect. 4.1.1).

We now assume *G* can be represented by some layout ${\mathcal {R}}$. For every *j*, $1\le j \le m$, we define $L_j = \{l^1_{j}, l^2_{j}, l^3_{j}\}$, for every *i*, $1 \le i \le n$, we define $A_{i} = \{t_i, f^1_i, f^2_i\}$, and, for every *j*, $1 \le j \le m-1$, we define $C^l_{j} = \{c_j, c_{j-1}, c^1_{j-1}, c^2_{j-1}\}$, $C^r_{j} = \{c_j, c_{j+1}, c^1_j, c^2_j\}$, and $C_j = C^l_j \cup C^r_j$.

The road map for the proof is as follows. We first consider the neighbourhood of $c_j$ and once we have fixed the layout for this subgraph, the structure of the whole layout can be concluded inductively. The closed neighbourhood of $c_j$ consists of $C^l_{j}$ and $C^r_{j}$ (two $K_4$ joined by $c_j$) and $L_j$, where all vertices of the two $K_4$ (except $c_j$) are not connected to any vertex of $L_j$. Intuitively speaking, this independence between $L_j$ and the $K_4$ of the backbone will force the backbone to expand along one dimension, say horizontally (as depicted in Fig. 10), while the visibilities between $L_j$ and $c_j$ must then be vertical (as depicted in Fig. 11(a)). However, formally proving this turns out to be quite complicated.

**Fig. 12:**
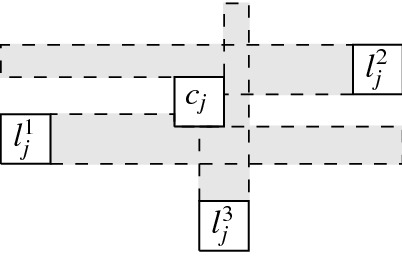
Possible placement of selectables for $c_j$

The general proof idea is to somehow place the unit squares $R_{L_{j}}$ in such a way that they all see $R_{c_j}$, for example, as depicted in Fig. 12 (in fact, this is one of the configurations that is not possible, since it does not leave enough space for all unit squares $R_{C_{j}}$). The grey area represents areas of visibility of the unit squares $R_{L_{j}}$. If we were able to show that no unit squares from $R_{C_{j}}$ can intersect the grey area, then this considerably restricts the possibilities to place the unit squares $R_{C_j}$ and by applying arguments of this type, it can be concluded, by exhaustively searching all possibilities and under application of Lemma 4.5, that the only possible layouts have the above described form. If a unit square $R_x$ from $R_{C_j\setminus \{c_j\}}$ is placed in this grey area, then either there is a visibility between this unit square and a unit square from $R_{L_{j}}$, which is not allowed as there are no edges between the associated vertices, or this forbidden visibility is blocked by other unit squares. This type of blocking would require a path between *x* and $c_j$ or some vertex from $L_j$; a neighbourhood-structure which, unfortunately, does exist in *G*. Consequently, in order to apply the above described argument, we first have to show that the existence of such visibility-blocking unit squares leads to a contradiction. However, before we are able to do this, we need to make a few more assumptions about the structure of the formula and we have to prove two technical lemmata.

Without loss of generality (if necessary with additional satisfiable clauses over new variables), we can assume that the clauses $c_1,\dots ,c_m$ are ordered so that, for each *i*, the indices

$$\begin{aligned} {\text {prev}}(y_{i,h})&:=\max {\{\{-6\} \cup \{j<i\mid c_j \text { contains } y_{i,h} \text { as literal}\}\}},\\ {\text {succ}}(y_{i,h})&:=\min {\{\{m+6\} \cup \{j>i\mid c_j \text { contains } y_{i,h} \text { as literal}\}\}} \end{aligned}$$

for $h=1,2,3$ differ from *i* and from each other for different values of *h* by at least six. We also need some more notations. Let $R_i,R_j,R_k$ be unit squares. If some visibility rectangle for $R_i$ and $R_k$ intersects $R_j$, then $R_j$ is *strictly between* $R_i$ and $R_k$; if this holds for *every* visibility rectangle for $R_i$ and $R_k$, then $R_j$ *blocks the view* between $R_i$ and $R_k$.

##### Lemma 4.7

For any $1\le j\le m$, no unit square for a vertex in $N(\overset{_{\leftarrow }}{t_j})\setminus \{h_{t_j}^0,\dots ,h_{t_j}^4\}$ can be vertically aligned with the unit square of a neighbour from $N(\overset{_{\leftarrow }}{t_j})\cup \{\overset{_{\leftarrow }}{t_j},\overset{_{\rightarrow }}{t_j}\}$ so that there is no unit square with smaller *x*-coordinate strictly between them.

##### Proof

**Fig. 13:**
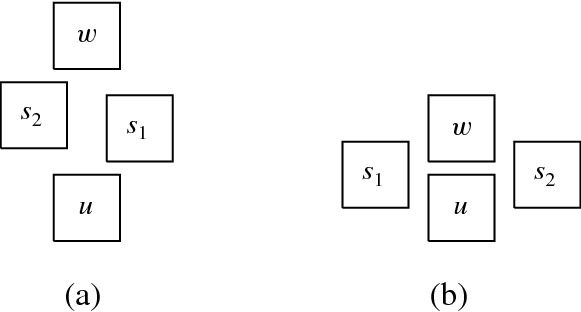
Illustrations for Lemma 4.7

Observe generally that for two vertically aligned squares $R_{u}, R_{w}$ there are only the two layouts, shown in Fig. 13, to place squares for two common neighbours $s_1,s_2$ of *u* and *w*. In Fig. 13(a) there is no possibility to avoid placing either $R_{s_1}$ or $R_{s_2}$ strictly between $R_{u}$ and $R_{w}$ with smaller *x*-coordinate. In Fig. 13(b), there is no possibility for a vertex $s_3$ adjacent to both $s_1$ and $s_2$ to place its square $R_{s_3}$ so that it sees both $R_{s_1}$ and $R_{s_2}$. We show that this neighbourhood structure among $s_1,s_2,s_3$, *u* and *w* occurs for any choice of $u\in N(\overset{_{\leftarrow }}{t_j})\setminus \{h_{t_j}^0,\dots ,h_{t_j}^4\}$ and $w\in N(u)\cap (N(\overset{_{\leftarrow }}{t_j})\cup \{\overset{_{\leftarrow }}{t_j},\overset{_{\rightarrow }}{t_j}\})$.

The neighbourhood $N(\overset{_{\leftarrow }}{t_j})$ is by definition $\{h_{t_j}^1,h_{t_j}^2,l^{r_1}_{j_1}, l^{r_2}_{j_2}, \ldots , l^{r_{q}}_{j_{q}},$ $h_{t_j}^0,t_j,h_{t_j}^3,h_{t_j}^4\}$ with $j_1< j_2< \ldots < j_q$ which builds a path in this order. If *w* is not in $\{\overset{_{\leftarrow }}{t_j},\overset{_{\rightarrow }}{t_j}\}$ the vertices $\overset{_{\leftarrow }}{t_j}$ and $\overset{_{\rightarrow }}{t_j}$ are the common neighbours $s_1$ and $s_2$ of *u* and *w*, while $h_{t_j}^1$ or $h_{t_j}^4$ can be considered as their common neighbour $s_3$. If $w\in \{\overset{_{\leftarrow }}{t_j},\overset{_{\rightarrow }}{t_j}\}$, the two vertices in $N(u)\setminus \{\overset{_{\leftarrow }}{t_j},\overset{_{\rightarrow }}{t_j}\}$ (observe that with the additional vertices $h_{t_j}^0,\dots ,h_{t_j}^4$, *u* always has exactly two neighbours in $N(\overset{_{\leftarrow }}{t_j})$) are the common neighbours $s_1$ and $s_2$ of *u* and *w*, while the vertex in $\{\overset{_{\leftarrow }}{t_j},\overset{_{\rightarrow }}{t_j}\}\setminus \{w\}$ is their common neighbour $s_3$. $\square$

##### Lemma 4.8

For all $1\le i\le m$ and $r\in \{1,2,3\}$ and $z\in N(c_i) \setminus \{l_i^r\}$, there is no path between $l_i^r$ and *z* which does not include $c_i$ such that the associated unit squares are vertically aligned and such that there exists no other unit square which is strictly between two unit squares of this path with strictly smaller *x*-coordinate.

##### Proof

Assume that there is such a path *P*. Regardless whether $z = l_i^t$ for some $t\in \{1,2,3\}\setminus \{r\}$ or $z\in C_{i} \setminus \{c_i\}$, *P* has to start with some neighbour of $l_i^r$. Let $y_{i,r}=x_j$ and let $N(\overset{_{\leftarrow }}{t_j})=\{h_{t_j}^1,h_{t_j}^2,l^{r_1}_{j_1}, l^{r_2}_{j_2}, \ldots , l^{r_{q}}_{j_{q}},h_{t_j}^0,t_j,h_{t_j}^3,h_{t_j}^4\}$ with $j_1< j_2< \ldots < j_q$ be the neighbourhood of $\overset{_{\leftarrow }}{t_j}$ which, by definition, builds a path in this order.

Since $y_{i,r}=x_j$, we can conclude that $l_{j_p}^{r_p}=l_i^r$, for some $p\in \{1,\dots ,q\}$. In order to reach $z\in N(c_i)$, the path *P* has to contain at least one vertex from $N(\overset{_{\leftarrow }}{t_j})\setminus \{h_{t_j}^0,\dots ,h_{t_j}^4\}$. By Lemma 4.7, it follows that there are only two options to choose a neighbour *u* of $l_{j_p}^{r_p}$ such that the path can then be continued with one of its neighbours *w* without violating the property that there is no unit square strictly between the unit squares for *u* and *w* with strictly smaller *x*-coordinate: either *P* starts with $l_{j_{p-1}}^{r_{p-1}}$ which then has to be followed by $c_{j_{p-1}}$ (if $p>1$) or $l_{j_{p+1}}^{r_{p+1}}$ which then has to be followed by $c_{j_{p+1}}$ (if $p<q$). Observe that all other neighbours of $l_{j_p}^{r_p}$ only have neighbours in $N(\overset{_{\leftarrow }}{t_j})\cup \{\overset{_{\leftarrow }}{t_j},\overset{_{\rightarrow }}{t_j}\}$ and that the only neighbours outside $N(\overset{_{\leftarrow }}{t_j})\cup \{\overset{_{\leftarrow }}{t_j},\overset{_{\rightarrow }}{t_j}\}$ for $l_{j_{p-1}}^{r_{p-1}}$ and $l_{j_{p+1}}^{r_{p+1}}$, are $c_{j_{p-1}}$ and $c_{j_{p+1}}$, respectively. Lemma 4.7 further implies that *P* contains no other selectable since each $l_s^t\in \{l_s^t\,|\,1\le s\le m,\,1\le t\le 3\}$ has only one neighbour outside $N(\overset{_{\leftarrow }}{t_{j'}})$ for some $j'$, continuing the path to *z* (recall $z\notin N(\overset{_{\leftarrow }}{t_{j'}})$ for any $j'$) however requires at least two such neighbours.

Consider, without loss of generality, that $p<q$ and that *P* contains $l_{j_{p+1}}^{r_{p+1}}$ followed by $c_{j_{p+1}}$. With the above definition of ${\text {succ}}$, we know that $j_{p+1}={\text {succ}}(y_{i,r})$. By the previously assumed properties of the input-formula, we know that $j_{p+1}$ differs from *i* and also from the smallest index $k>i$ for which $c_i$ shares a literal other than $y_{i,r}$ with $c_k$ by at least six; especially $c_i$ and $c_{j_{p+1}}$ share no common literal other than $x_j = y_{i, r}$. This means that *P* has to continue from $c_{j_{p+1}}$ with at least five vertices from $V_c\setminus \{l_s^t\,|\,1\le s\le m,\,1\le t\le 3\}$; observe that there are no other paths that avoid selectables.

**Fig. 14:**
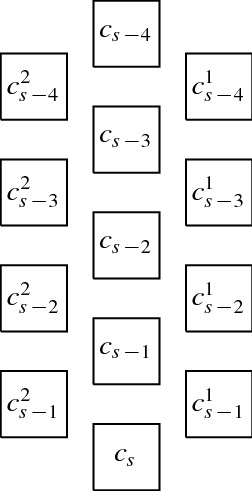
Illustration for the proof of Lemma 4.8

Let $s=j_{p+1}$. Assume that the vertex following $c_s$ on *P* is some $v\in C^l_s \setminus \{c_s\}$ (the case $v\in C^r_s\setminus \{c_s\}$ is analogous and these are the only non-selectable neighbours of $c_s$). The vertices $C^l_{s}$ build a $K_4$ and, by Lemma 4.5, the only possibility for a layout of this $K_4$ such that there is no unit square with smaller *x*-coordinate strictly between the vertically aligned $R_{v}$ and $R_{c_s}$, is case 1. from Fig. 8 with $R_{v}$ and $R_{c_s}$ taking the role of $R_2$ and $R_4$. Observe that in case 1. there is no possibility to add a unit square which sees both $R_1$ and $R_3$ without destroying any of the $K_4$ edges. The only possibility for *v* is hence $c_{s-1}$, since $c_{s-1}$ and $c_{s-1}^t$ have the common neighbour $c_{s-2}^t$ for $t=1,2$ which could not be placed otherwise. By the same argument, the whole part of at least five vertices from $V_c\setminus \{l_s^t\,|\,1\le s\le m,\,1\le t\le 3\}$ in *P* are in fact vertices in $\{c_j\,|\,1\le j\le m\}$ and especially contain the sequence $c_s,c_{s-1},c_{s-2},c_{s-3},c_{s-4}$ which has to be arranged as illustrated in Fig. 14.

There is no possibility to enable visibility representing the edge $\{c_{s-2},l_{s-2}^1\}$ so that $R_{l_{s-2}^1}$ sees none of the unit squares $R_{c_{s-2}^1},R_{c_{s-2}^2},R_{c_{s-3}^1},R_{c_{s-3}^2}$, for these unit squares however, all neighbours have been placed, so there is no possibility to block this unwanted visibility which overall yields a contradiction to the existence of *P*. $\square$

We are now ready to prove that no $R_x$ from $R_{C_j\setminus \{c_j\}}$ can be placed within visibility of the squares $R_{L_{j}}$ (in the sense of the discussion below Fig. 12, see also the figure itself). We state the corresponding lemma in a way that it is more applicable in the following proofs.

##### Lemma 4.9

For all *i*, $1\le i\le m$, $r\in \{1,2,3\}$, and every $R_z$ with $z\in N(c_i)\setminus \{l_i^r\}$, there exists no non-degenerate axis-parallel rectangle *S* which is not intersected by $R_{c_i}$ such that one side of *S* is in $R_{l_i^r}$ and the opposite side is in $R_z$. In particular, this implies the following properties:

1. $R_z$ is not strictly between $R_{c_i}$ and $R_{l_i^r}$.
2. $R_{l_i^r}$ is not strictly between $R_{c_i}$ and $R_z$.
3. If $R_{c_i}$ is strictly between $R_{l_i^r}$ and $R_z$ then $R_{c_i}$ blocks the view between $R_{l_i^r}$ and $R_z$.

##### Proof

**Fig. 15:**
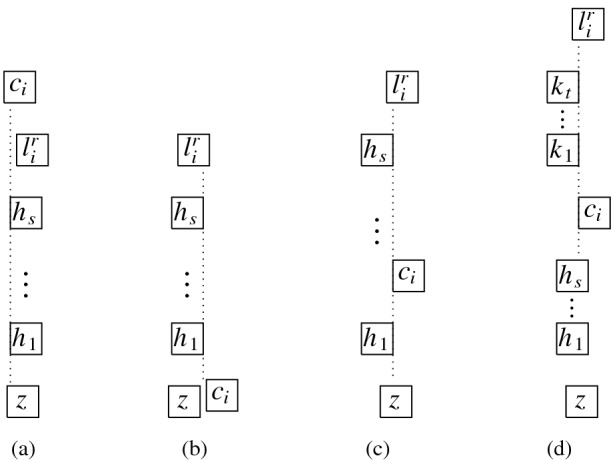
Illustrations for the proof of Lemma 4.9

Let *z*, *i* and *r* be as in the statement of the lemma. We assume that there exists a non-degenerate axis-parallel rectangle *S* which is not intersected by $R_{c_i}$ such that one side of *S* is in $R_{l_i^r}$ and the opposite side is in $R_z$. Without loss of generality, we assume that, after removing all unit squares except $R_{l_i^r}$ and $R_{z}$ from the layout, we have $R_{l_i^r} {{\,\mathrm{\downarrow }\,}} R_{z}$, and, furthermore, that the *x*-coordinate of $R_{l_i^r}$ is not smaller than the *x*-coordinate of $R_z$ (so we have the situation shown in Fig. 15). Moreover, since *z* is not adjacent to $l_i^r$, some further unit square(s) have to block the visibility (otherwise implied by the rectangle *S*) between $R_{l_i^r}$ and $R_z$, while $R_{c_i}$ has to see both $R_z$ and $R_{l_i^r}$. There are only the following possibilities for this situation:

1. $R_{c_i}{{\,\mathrm{\downarrow }\,}} \{R_{l_i^r},R_z\}$ or $\{R_{l_i^r},R_z\}{{\,\mathrm{\downarrow }\,}} R_{c_i}$ (see Fig. 15(a)): We only consider the case $R_{c_i}{{\,\mathrm{\downarrow }\,}}\{R_{l_i^r},R_z\}$, since $\{R_{l_i^r},R_z\}{{\,\mathrm{\downarrow }\,}} R_{c_i}$ can be dealt with analogously. Let $R_{h_1},\dots ,R_{h_s}$ be the unit squares strictly between $R_{l_i^r}$ and $R_z$ (to intersect *S* in order to block the view) of minimum *x*-coordinate sorted by *y*-coordinate. Observe that each $R_{h_t}$ has a larger *x*-coordinate than $R_{z}$ since otherwise there is no visibility between $R_{c_i}$ and $R_z$. If there is a unit square *R* strictly between some $R_{h_t}$ and $R_{h_{t+1}}$ with smaller *x*-coordinate, then this either contradicts the definition of the $R_{h_t}$ (i.e., if *R* is strictly between $R_{l_i^r}$ and $R_z$) or, again, the visibility between $c_i$ and *z* would be blocked. Consequently, there is no such unit square strictly between some $R_{h_t}$ and $R_{h_{t+1}}$ with smaller *x*-coordinate.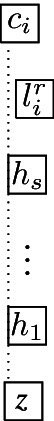 The vertices $h_1,\dots ,h_s$ corresponding to $R_{h_1},\dots ,R_{h_s}$ hence describe a path which does not include $c_i$ and for which $h_1$ is adjacent to $z\in N(c_i)\setminus \{l_i^r\}$ and $h_s$ is adjacent to $l_i^r$. By their choice as the squares strictly between $R_{l_i^r}$ and $R_z$ with minimum *x*-coordinate, the unit squares $R_{h_1},\dots ,R_{h_s}$ are aligned and no unit square with smaller *x*-coordinate is strictly between any $R_{h_t}$ and $R_{h_{t+1}}$ which is a contradiction to Lemma 4.8.
2. ($R_z{{\,\mathrm{\rightarrow }\,}}R_{c_i}$ and $R_{l_i^r}{{\,\mathrm{\downarrow }\,}} R_{c_i}$) or ($R_{c_i}{{\,\mathrm{\downarrow }\,}} R_z$ and $R_{c_i}{{\,\mathrm{\rightarrow }\,}}R_{l_i^r}$) (see Fig. 15(b)): We only consider the case ($R_z{{\,\mathrm{\rightarrow }\,}}R_{c_i}$ and $R_{l_i^r}{{\,\mathrm{\downarrow }\,}} R_{c_i}$), since the case ($R_{c_i}{{\,\mathrm{\downarrow }\,}} R_z$ and $R_{c_i}{{\,\mathrm{\rightarrow }\,}}R_{l_i^r}$) can be dealt with analogously. To preserve the visibility $R_{l_i^r}{{\,\mathrm{\downarrow }\,}} R_{c_i}$, all unit squares which intersect *S* must have an *x*-coordinate strictly smaller than $R_{l_i^r}$. Let $R_{h_1},\dots ,R_{h_s}$ be the unit squares strictly between $R_{l_i^r}$ and $R_z$ (to intersect *S* in order to block the view) of maximum *x*-coordinate. These unit squares have the same properties as for case 1. (with “strictly larger” instead of “strictly smaller”), which yields a contradiction to Lemma 4.8.
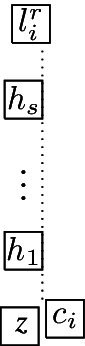
3. $R_{c_i}{{\,\mathrm{\downarrow }\,}} R_z$ and $R_{l_i^r}{{\,\mathrm{\downarrow }\,}} R_{c_i}$ (see Fig. 15, (c) and (d)): Note that *S* is not intersected by $R_{c_i}$ and, without loss of generality, we assume that *S* lies to the left of $R_{c_i}$. Among all unit squares which intersect *S*, let $R_{h_1},\dots ,R_{h_s}$ be the ones of maximum *x*-coordinate, sorted by *y*-coordinate. We consider two different cases according to whether one of the unit squares $R_{h_1},\dots ,R_{h_s}$ sees $R_{c_i}$ vertically or not:
  - We assume that there is no *j*, $1\le j\le s$, such that $R_{h_j}$ sees $R_{c_i}$ vertically (see Fig. 15(c)). This implies that the vertices associated to $R_{h_1},\dots ,R_{h_s}$ build a path from *z* to $l_i^r$ and $c_i$ is not included in this path (observe that although one or even two of the vertices $h_1,\dots ,h_s$ could be neighbours of $c_j$ by horizontal visibility, vertex $c_j$ itself is not among the vertices $h_1,\dots ,h_s$). Further, since the *x*-coordinate of the unit squares $R_{h_1},\dots ,R_{h_s}$ is assumed to be maximum among the unit squares which intersect *S*, there is no other unit square which lies strictly between some $R_{h_t}$ and $R_{h_{t+1}}$ and has a larger *x*-coordinate. Since $R_{h_1},\dots ,R_{h_s}$ all have the same *x*-coordinate and are hence vertically aligned, this is a contradiction to Lemma 4.8.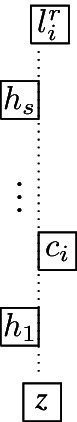  - We assume that, for some *j*, $1\le j\le s$, the unit square $R_{h_j}$ sees $R_{c_i}$ vertically (see Fig. 15(d)). This is only possible if this $R_{h_j}$ is between $R_{c_i}$ and either $R_{l_i^r}$ or $R_z$. However, if $R_{h_j}$ is between $R_{c_i}$ and $R_{l_i^r}$, then, with $R_{h_j}$ playing the role of *z* (note that $h_j \in N(c_i)\setminus \{l_i^r\}$), we obtain case 1. again. Thus, we can assume that $R_{h_j}$ is between $R_{c_i}$ and $R_z$. Furthermore, if $j < s$, then $R_{h_{j+1}}$ is between $R_{c_i}$ and $R_{l_i^r}$ and we obtain case 1. as before. Consequently, $j=s$ and $h_s\in N(c_i)$. Since $l_i^r$ is not adjacent to any neighbour of $c_i$, $l_i^r$ and $h_s$ are not adjacent; thus, the visibility between $R_{h_s}$ and $R_{l_i^r}$ must be blocked. Let $R_{k_1},\dots , R_{k_t}$ be the unit squares of maximum *x*-coordinate which intersect every visibility rectangle between $R_{h_s}$ and $R_{l_i^r}$, sorted by *y*-coordinate. We note that if they are strictly between $R_{l^r_i}$ and $R_{c_i}$, then we obtain case 1. again, with $R_{k_1}$ playing the role of *z*. 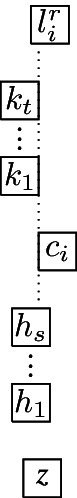
Moreover, if they have an *x*-coordinate that is more than one less than the *x*-coordinate of $R_{c_i}$, then they would not block the visibility between $R_{h_s}$ and $R_{l_i^r}$. Consequently, these unit squares all have the same *x*-coordinate which is exactly one less than the *x*-coordinate of $c_i$ (as shown in Fig. 15(d)). The vertices associated to $R_{k_1},\dots , R_{k_t}$ again build a path between $l_i^r$ and some neighbour of $c_i$ and do not include $c_i$, and, as explained above, there is no unit square strictly between some $R_{k_t}$ and $R_{k_{t+1}}$ of larger *x*-coordinate. Hence, the path $R_{k_1},\dots , R_{k_t}$ is also a contradiction to Lemma 4.8.

Since Lemma 4.8 holds equivalently, the same argumentation yields this result for $t_j$ or $f_j^1$ or $f_j^2$ instead of $l_i^r$, and $x_j$ instead of $c_i$ for all $1\le j\le n$. $\square$

With the help of Lemma 4.9, we can now apply the argument sketched below Fig. 12 in order to show that $R_{C^l_{j}}$ and $R_{C^r_{j}}$ cannot all see $R_{c_j}$ from the same side, which can then be used to show that either all $R_{L_j}$ see $R_{c_j}$ vertically or all of them see $R_{c_j}$ horizontally (see Lemma 4.11):

##### Lemma 4.10

For every *j*, $1 \le j \le m - 1$ and $y \in C_j \setminus \{c_j\}$, $R_{c_j} {{\,\mathrm{\rightarrow }\,}}R_{C_j \setminus \{y, c_j\}}$ is not possible.

**Fig. 16:**
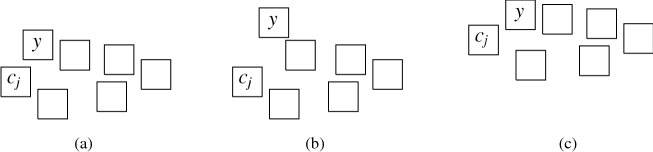
Illustrations for the proof of Lemma 4.10

##### Proof

We first note that, independent from the choice of *y*, either $C^l_j$ or $C^r_j$ is completely contained in $R_{C_j \setminus \{y\}}$. We assume that the former applies, which means that the $K_4$ on vertices $C^l_j$ must satisfy case 1. of Lemma 4.5 with $c_j$ playing the role of $R_1$, or it satisfies case 3. of Lemma 4.5 with $c_j$ playing the role of $R_4$. Next, we note that $R_y {{\,\mathrm{\rightarrow }\,}}R_{c_j}$ is not possible, since then the $K_4$ on vertices $C^r_j$ contains a unit square, namely $R_{c_j}$, which horizontally sees all other vertices, but not in the same direction and this is, according to Lemma 4.5, not possible; thus, we have either case $R_y{{\,\mathrm{\updownarrow }\,}} R_{c_j}$ or case $R_{c_j} {{\,\mathrm{\rightarrow }\,}}R_y$, which we shall now consider separately.

**Fig. 17:**
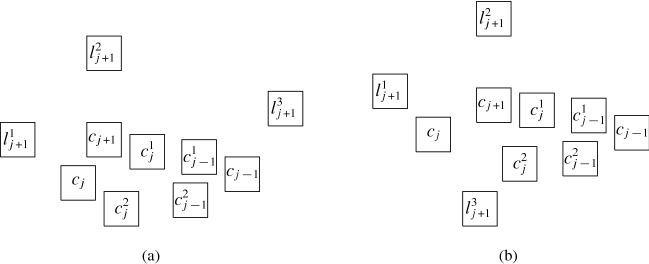
Illustrations for the proof of Lemma 4.10

1. Case $R_y{{\,\mathrm{\downarrow }\,}} R_{c_j}$ (the case $R_{c_j}{{\,\mathrm{\downarrow }\,}} R_y$ can be handled analogously): For the $K_4$ on vertices $C^r_{j}$, we have that $R_y{{\,\mathrm{\downarrow }\,}} R_{c_j}$, while all unit squares $R_{C^r_{j}\setminus \{c_j, y\}}$ see $R_{c_j}$ horizontally to the same side. This means that the $K_4$ on vertices $C^r_{j}$ satisfies case 2. or case 3. of Lemma 4.5. If it satisfies case 2., then we have the situation illustrated in Fig. 16(a) (where the four vertices on the left are the vertices from $C^r_{j}$). On the other hand, if it satisfies case 3., then we claim that the only unit square that can play the role of the unit square $R_4$ (i.e., the one that sees all the others by the same kind of visibility) is $R_{y}$. In order to verify this claim, we first observe that $R_{c_j}$ cannot play the role of $R_4$, since it sees $R_{y}$ vertically and the two other unit squares in $R_{C^r_{j}\setminus \{c_j, y\}}$ horizontally. If, for a $z \in C^r_j \setminus \{y, c_j\}$, $R_z$ plays the role of $R_4$, then, since $R_{c_j}{{\,\mathrm{\rightarrow }\,}}R_z$, we must have $R_{C^r_j\setminus \{z\}} {{\,\mathrm{\rightarrow }\,}}R_{z}$; in particular, $R_{z}$ plays the role of $R_4$, $R_{c_j}$ plays the role of $R_2$ and $R_{y}$ plays the role of $R_1$. Now it is not possible to add another unit square *R* with $R_{c_j}{{\,\mathrm{\rightarrow }\,}}R$, since in order to see $R_{c_j}$, *R* must be placed to the left of $R_{z}$, which means that it necessarily blocks the visibility between $R_{z}$ and one of $C^r_j \setminus \{c_j, z\}$. This is a contradiction, since such a unit square must exist in order to represent the edges of the $K_4$ on vertices $C^l_{j}$. Consequently, if the $K_4$ on vertices $C^r_{j}$ satisfies case 3., then we have the situation illustrated in Fig. 16(b). We now turn to the $K_4$ on vertices $R_{C^l_{j}}$. As mentioned before, the relation $R_{c_j} {{\,\mathrm{\rightarrow }\,}}R_{C^l_{j} \setminus \{c_j\}}$ implies that the $K_4$ on vertices $C^l_{j}$ must satisfy case 1. of Lemma 4.5 with $R_{c_{j}}$ playing the role of $R_1$ or case 3. of Lemma 4.5 with $R_{c_j}$ playing the role of $R_4$. (Note that in Fig. 16, (a) and (b), we only illustrate case 1. for the $K_4$ on vertices $C^l_{j}$, which are the three vertices to the right together with vertex $c_j$; the following arguments can be carried out analogously for the case that the $K_4$ on vertices $C^l_{j}$ satisfies case 3. of Lemma 4.5.) However, for both situations depicted in Fig. 16, (a) and (b), it is not possible that a unit square in $R_{C^l_{j} \setminus \{c_j\}}$ sees $R_{c_j}$ horizontally and at the same time (by any kind of visibility) also $R_{y}$. Thus, *y* must be a vertex that is not connected to any vertex from $R_{C^l_{j} \setminus \{c_j\}}$, which implies $y = c_{j + 1}$. More precisely, since $R_{c^1_{j-1}} \in R_{C^l_{j} \setminus \{c_j\}}$ must see both $R_{c_j}$ and $R_{c^1_{j}}$, and $R_{c^2_{j-1}} \in R_{C^l_{j} \setminus \{c_j\}}$ must see both $R_{c_j}$ and $R_{c^2_{j}}$, we can conclude $y \notin \{c^1_{j}, c^2_{j}\}$, which implies $y = c_{j + 1}$.
  - Situation illustrated in Fig. 16(b) (repeated on the left):
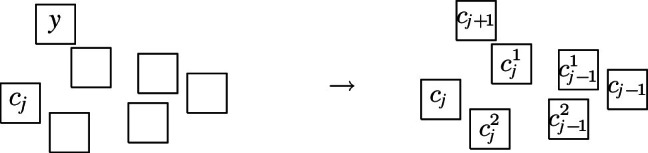
 We assume that $R_{c^1_{j}}{{\,\mathrm{\downarrow }\,}} R_{c^2_{j}}$ (note that, due to the edges $\{c^1_{j}, c^1_{j-1}\}$ and $\{c^2_{j}, c^2_{j-1}\}$, this uniquely defines all the unlabeled unit squares of Fig. 16(b)); the case $R_{c^2_{j}}{{\,\mathrm{\downarrow }\,}} R_{c^1_{j}}$ can be handled analogously. We now consider the vertices $c^1_{j+1}$ and $c^2_{j+1}$ from the $K_4$ on vertices $C^l_{j + 1}$ (which we have not considered so far and which are not present in Fig. 16(b)). The vertex $c^1_{j+1}$ is connected to both $c_{j + 1}$ and $c^1_{j}$ and, likewise, the vertex $c^2_{j+1}$ is connected to both $c_{j + 1}$ and $c^2_{j}$. To see both $R_{c_{j + 1}}$ (the square labelled *y* in Fig. 16(b)) and $R_{c^2_{j}},R_{c^2_{j+1}}$ must be placed so that $R_{c_{j + 1} } {{\,\mathrm{\downarrow }\,}} R_{c^2_{j+1}}$ and $R_{c^2_{j+1}}{{\,\mathrm{\rightarrow }\,}}R_{c_{j}^2}$ or $R_{c_{j}^2}{{\,\mathrm{\downarrow }\,}} R_{c_{j+1}^2}$ (note that $R_{c_{j}^2}{{\,\mathrm{\downarrow }\,}} R_{c^2_{j+1}}$ would block the visibility of $R_{c_{j + 1} }$ and either $R_{c_{j}}$ or $R_{c^1_{j} }$). This means that $R_{c^2_{j+1}}$ is placed below $R_{c_{j}}$ which means that the *y*-coordinates of $R_{c^2_{j+1}}$ and $R_{c_{j+1}}$ differ by more than one. Further, there are two rectangles strictly between $R_{c^2_{j+1}}$ and $R_{c_{j+1}}$ that require visibility to these two ($R_{c_j}$ and $R_{c_j^2}$) which leaves no possibility for $R_{c^1_{j + 1}}$ to see both $R_{c^2_{j+1}}$ and $R_{c_{j+1}}$ without blocking a visibility among the rectangles for $\{c_j,c_{j+1}, c_j^2,c_{j+1}^2\}$. This means that the situation illustrated in Fig. 16(b) is not possible.
  - Situation illustrated in Fig. 16(a):
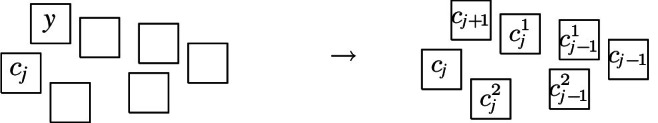
 Similarly as in the previous case, we assume that $R_{c^1_{j}}{{\,\mathrm{\downarrow }\,}} R_{c^2_{j}}$ (again, this uniquely defines all the unlabeled unit squares of Fig. 16(a)) and note that the case $R_{c^2_{j}}{{\,\mathrm{\downarrow }\,}} R_{c^1_{j}}$ can be handled analogously. We now consider all possibilities of how the unit squares $R_{L_{j + 1}}$ for the selectables $l^1_{j +1},l^2_{j + 1}, l^3_{j + 1}$ can be placed so that they see $R_{c_{j + 1}}$ without other unwanted visibilities. If, for some *r*, $1\le r \le 3$, $R_{c_{j+1}}{{\,\mathrm{\downarrow }\,}} R_{l^r_{j+1}}$, then there is at least one unit square $R_{z}$, with $z \in N(c_{j + 1})$, strictly between $R_{c_{j+1}}$ and $R_{l^r_{j+1}}$, which according to case 1. of Lemma 4.9, is not possible. If, for some *r*, $1 \le r \le 3$, $R_{l^r_{j+1}}{{\,\mathrm{\downarrow }\,}} R_{c_{j+1}}$, so that $R_{l^r_{j+1}}$ and $R_{c_{j+1}}$ are not aligned, then there is at least one unit square $R_{z}$, with $z \in N(c_{j + 1})$, such that $R_{c_{j + 1}}$ does not block the view between $R_{l_{j+1}^r}$ and $R_z$, which according to case 3. of Lemma 4.9, is not possible. Consequently, there is at most one *r*, $1\le r\le 3$, such that $R_{l^r_{j+1}}{{\,\mathrm{\downarrow }\,}} R_{c_{j+1}}$ and, furthermore, $R_{l^r_{j+1}}$ and $R_{c_{j+1}}$ must be aligned. If, for some *r*, $1 \le r \le 3$, $R_{c_{j+1}} {{\,\mathrm{\rightarrow }\,}}R_{l^r_{j+1}}$, then, due to case 1. of Lemma 4.9 and the position of $R_{c^1_j}$, we can conclude that $R_{l^r_{j+1}}$ is not aligned with $R_{c_{j+1}}$, but shifted upwards. Furthermore, again due to case 1. of Lemma 4.9, there is at most one such $R_{l^r_{j+1}}$ with $R_{c_{j+1}} {{\,\mathrm{\rightarrow }\,}}R_{l^r_{j+1}}$. If, for some *r*, $1 \le r \le 3$, $R_{l^r_{j+1}} {{\,\mathrm{\rightarrow }\,}}R_{c_{j+1}}$, such that $R_{l^r_{j+1}}$ and $R_{c_{j+1}}$ are not aligned, but $R_{l^r_{j+1}}$ is shifted downwards, $R_{c_{j + 1}}$ does not block the view between $R_{c_j^1}$ and $R_{l_{j+1}^r}$ which according to case 3. of Lemma 4.9, is not possible. In particular, due to case 1. of Lemma 4.9, this means that there is at most one $R_{l^r_{j+1}}$ with $R_{l^r_{j+1}} {{\,\mathrm{\rightarrow }\,}}R_{c_{j+1}}$, which is either aligned with $R_{c_{j+1}}$ or shifted upwards. However, we may assume that there is an *r*, $1\le r\le 3$, with $R_{c_{j+1}} {{\,\mathrm{\rightarrow }\,}}R_{l^r_{j+1}}$ (which, as explained above, is shifted upwards), since otherwise not all three unit squares in $R_{L_{j + 1}}$ can be placed. Consequently, by applying case 3. of Lemma 4.9, if there is an $R_{l^r_{j+1}}$ with $R_{l^r_{j+1}} {{\,\mathrm{\rightarrow }\,}}R_{c_{j+1}}$, then $R_{l^r_{j+1}}$ is aligned with $R_{c_{j+1}}$. We conclude that the unit squares in $R_{L_{j + 1}}$ must be placed as illustrated in Fig. 17(a) (obviously, the positions of the unit squares in $R_{L_{j + 1}}$ can be switched). Now consider the unit squares $R_{c^1_{j + 1}}$ and $R_{c^2_{j + 1}}$ from $C^l_{j + 1}$, which both must see $R_{c_{j + 1}}$. Due to the positions of $R_{l^1_{j + 1}}$ and $R_{l^2_{j + 1}}$, and due to cases 1. and 2. of Lemma 4.9, for every $z \in \{c^1_{j + 1}, c^2_{j + 1}\}$, neither $R_{z} {{\,\mathrm{\rightarrow }\,}}R_{c_{j + 1}}$ nor $R_{z}{{\,\mathrm{\downarrow }\,}} R_{c_{j + 1}}$ is possible. If $R_{c_{j + 1}} {{\,\mathrm{\rightarrow }\,}}R_{c^2_{j + 1}}$, then, in order to also see $R_{c^2_{j}}$, $R_{c^2_{j + 1}}$ must be placed so that $R_{c^2_{j + 1}} {{\,\mathrm{\downarrow }\,}} R_{c^2_{j}}$; as there is no space between $R_{c^2_{j + 1}}$ and $R_{c^1_{j}}$ to add another unit square, this implies that $R_{c^2_{j + 1}} {{\,\mathrm{\downarrow }\,}} R_{c^1_{j}}$ without enough space to block this unwanted visibility, which is a contradiction. Consequently, $R_{c_{j + 1}} {{\,\mathrm{\downarrow }\,}} R_{c^2_{j + 1}}$. Clearly, $R_{c_{j + 1}} {{\,\mathrm{\downarrow }\,}} R_{c^1_{j + 1}}$ is not possible, since then visibility between $R_{c^1_{j + 1}}$ and $R_{c^1_{j}}$ is not possible. Hence, $R_{c_{j + 1}} {{\,\mathrm{\rightarrow }\,}}R_{c^1_{j + 1}}$ (recall that above we have excluded all other directions). However, now there is no visibility between $R_{c^1_{j + 1}}$ and $R_{c^2_{j + 1}}$, which is a contradiction. Consequently, the situation illustrated in Fig. 16(a) is not possible.
2. Case $R_{c_j} {{\,\mathrm{\rightarrow }\,}}R_y$: We first note that this yields the situation illustrated in Fig. 16(c).
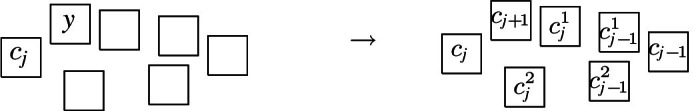
 Again, we only consider the situation where the $K_4$ on $C^l_j$ satisfies case 1. of Lemma 4.5 as the further argument does not differ for case 3. of Lemma 4.5. In the same way as for case 1. from above, we can conclude that $y = c_{j+1}$. We again assume $R_{c^1_{j}} {{\,\mathrm{\downarrow }\,}} R_{c^2_{j}}$ (since $R_{c^2_{j}}{{\,\mathrm{\downarrow }\,}} R_{c^1_{j}}$ can be handled analogously), we note that this uniquely defines all unit squares (as illustrated in Fig. 17(b)), and again we consider how the selectables for $c_{j+1}$ can be placed in order to see $R_{c_{j + 1}}$. Note that from case 1. of Lemma 4.9, it follows that if, for some *r*, $1\le r\le 3$, $R_{c_{j+1}} {{\,\mathrm{\leftrightarrow }\,}}R_{l^r_{j+1}}$, then $R_{l^r_{j+1}}$ is not aligned with $R_{c_{j+1}}$, but shifted upwards. Moreover, by case 3. of Lemma 4.9, there is at most one *r*, $1 \le r \le 3$, with $R_{c_{j+1}} {{\,\mathrm{\leftrightarrow }\,}}R_{l^r_{j+1}}$. From case 3. of Lemma 4.9 it also follows that if, for some *r*, $1 \le r \le 3$, $R_{l^r_{j+1}}{{\,\mathrm{\downarrow }\,}} R_{c_{j+1}}$, then $R_{l^r_{j+1}}$ is aligned with $R_{c_{j+1}}$ or shifted to the left. From case 1. of Lemma 4.9 it follows that if, for some *r*, $1\le r \le 3$, $R_{c_{j+1}}{{\,\mathrm{\downarrow }\,}} R_{l^r_{j+1}}$, then $R_{l^r_{j+1}}$ is not aligned with $R_{c_{j+1}}$, but shifted to the left. Since, according to case 3. of Lemma 4.9, it is not possible that, for some $r,r'$, $1\le r<r'\le 3$, $R_{l^r_{j+1}}{{\,\mathrm{\downarrow }\,}} R_{c_{j+1}}$ and $R_{c_{j+1}} {{\,\mathrm{\downarrow }\,}} R_{l^{r'}_{j+1}}$ in such a way that both $R_{l^{r}_{j+1}}$ and $R_{l^{r'}_{j+1}}$ are not aligned with $R_{c_{j+1}}$, but shifted to the left, we can conclude that we either have the situation illustrated in Fig. 17(b), or a similar situation with the only difference that $R_{c_{j + 1}} {{\,\mathrm{\rightarrow }\,}}R_{l^1_{j + 1}}$ instead of $R_{l^1_{j + 1}} {{\,\mathrm{\rightarrow }\,}}R_{c_{j + 1}}$, which can be handled analogously. Again, we now consider the unit squares $R_{c^1_{j + 1}}$ and $R_{c^2_{j + 1}}$. We first note that, due to cases 1. and 2. of Lemma 4.9, $R_{c^1_{j+1}}{{\,\mathrm{\downarrow }\,}} R_{c_{j + 1}}$ is not possible. If $R_{c^1_{j + 1}} {{\,\mathrm{\rightarrow }\,}}R_{c_{j + 1}}$, then the position of $R_{l^1_{j + 1}}$ and cases 1. and 2. of Lemma 4.9 imply that $R_{c^1_{j + 1}}$ has a *y*-coordinate not larger than $R_{c_{j + 1}}$. However, then either $R_{c^1_{j + 1}}$ cannot see $R_{c^1_{j}}$, or it blocks the view between $R_{c_j}$ and $R_{c^1_{j}}$. If $R_{c_{j +1}} {{\,\mathrm{\downarrow }\,}} R_{c^1_{j+ 1}}$, then, due to the position of $R_{l^3_{j+1}}$ and cases 1. and 2. of Lemma 4.9, $R_{c^1_{j + 1}}$ cannot be aligned with $R_{c_{j+1}}$, but must be shifted to the right. However, then $R_{c^1_{j + 1}}$ cannot see $R_{c^1_{j}}$. Consequently, we can conclude that $R_{c_{j + 1}} {{\,\mathrm{\rightarrow }\,}}R_{c^1_{j + 1}}$. In the same way as above, we can exclude $R_{c^2_{j + 1}}{{\,\mathrm{\downarrow }\,}} R_{c_{j + 1}}$ and $R_{c^2_{j + 1}} {{\,\mathrm{\rightarrow }\,}}R_{c_{j + 1}}$. If $R_{c_{j + 1}}{{\,\mathrm{\downarrow }\,}} R_{c^2_{j+1}}$, then, due to $R_{c^2_j}$, $R_{c^2_{j + 1}}$ cannot see $R^1_{c_{j + 1}}$. Consequently, we must have $R_{c_{j + 1}} {{\,\mathrm{\rightarrow }\,}}R_{c^2_{j + 1}}$. However, there is no way for $R_{c^2_{j+1}}$ to see both $R_{c_{j + 1}}$ and $R_{c^2_j}$ without seeing $R_{c^1_j}$ (i.e., $R_{c^2_{j+1}}$ would have been placed with less than one unit distance to $R_{c^1_j}$). Consequently, the situation illustrated in Fig. 16(b) is not possible. $\square$

Lemmas 4.5, 4.9 and 4.10 can now be used to show that in fact the unit squares from $R_{L_j}$, representing the literals (i.e., the selectables) for the $j^{\text {th}}$ clause gadget, see $R_{c_j}$ all either horizontally or vertically.

##### Lemma 4.11

For every *j*, $1 \le j \le m$, either $R_{c_j} {{\,\mathrm{\leftrightarrow }\,}}R_{L_j}$ or $R_{c_j}{{\,\mathrm{\updownarrow }\,}} R_{L_j}$.

##### Proof

We first observe that it is not possible that $R_{c_j} {{\,\mathrm{\rightarrow }\,}}R_{L_j}$, $R_{L_j} {{\,\mathrm{\rightarrow }\,}}R_{c_j}$, $R_{c_j}{{\,\mathrm{\downarrow }\,}} R_{L_j}$ or $R_{L_j}{{\,\mathrm{\downarrow }\,}} R_{c_j}$. More precisely, all these cases mean that there are $R, R' \in R_{L_j}$, such that $R'$ is strictly between *R* and $R_{c_j}$, which is a contradiction to case 1. of Lemma 4.9. Hence we only have to rule out the following cases (for the sake of convenience, we set $R_{L_j} = \{x, y, z\}$):

1. $$x {{\,\mathrm{\downarrow }\,}} R_{c_j}$$, $$R_{c_j} {{\,\mathrm{\downarrow }\,}} y$$, and $$R_{c_j} {{\,\mathrm{\leftrightarrow }\,}}z$$,
2. $$x {{\,\mathrm{\rightarrow }\,}}R_{c_j}$$, $$R_{c_j} {{\,\mathrm{\rightarrow }\,}}y$$ and $$R_{c_j}{{\,\mathrm{\updownarrow }\,}} z$$,
3. $$\{x, y\} {{\,\mathrm{\downarrow }\,}} R_{c_j}$$ and $$R_{c_j} {{\,\mathrm{\leftrightarrow }\,}}z$$,
4. $$R_{c_j} {{\,\mathrm{\downarrow }\,}}\{x, y\}$$ and $$R_{c_j} {{\,\mathrm{\leftrightarrow }\,}}z$$,
5. $$\{x, y\} {{\,\mathrm{\rightarrow }\,}}R_{c_j}$$ and $$R_{c_j}{{\,\mathrm{\updownarrow }\,}} z$$,
6. $$R_{c_j} {{\,\mathrm{\rightarrow }\,}}\{x, y\}$$ and $$R_{c_j}{{\,\mathrm{\updownarrow }\,}} z$$.

Since cases 1. and 2. are symmetric, as well as cases 3., 4., 5., and 6., we only consider cases 1. and 3.

1. Case 1. ($$x {{\,\mathrm{\downarrow }\,}} R_{c_j}$$, $$R_{c_j} {{\,\mathrm{\downarrow }\,}} y$$, and $$R_{c_j} {{\,\mathrm{\leftrightarrow }\,}}z$$):
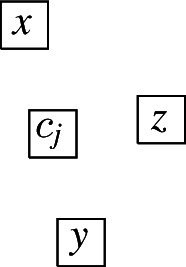
 We assume that $R_{c_j} {{\,\mathrm{\rightarrow }\,}}z$; the case $z {{\,\mathrm{\rightarrow }\,}}R_{c_j}$ can be handled analogously. Due to statement 3. of Lemma 4.9, we can assume that $R_{c_j}$ blocks the view between *x* and *y* (which, in particular, means that *x* and *y* cannot both be shifted to the same side w.r.t. $R_{c_j}$). We now consider the $K_4$ on vertices $C^l_{j}$. First, we assume that the unit squares $R_{C^l_{j}}$ are placed so that for some $S, S' \in R_{C^l_{j}}$ with $S \ne S'$, $R_{c_{j}} {{\,\mathrm{\rightarrow }\,}}\{S, S'\}$. By consulting Lemma 4.5, we observe that this means that there are $T, T' \in R_{C^l_{j}}$, such that $R_{c_{j}}{{\,\mathrm{\rightarrow }\,}}\{T, T'\}$, neither *T* nor $T'$ are aligned with $R_{c_{j}}$, *T* is shifted upwards and $T'$ is shifted downwards. However, this necessarily means that there is a unit square $R\in R_{N(c_j)}$ (*T* or $T'$), such that *R* is strictly between $R_{c_j}$ and *z*, or *z* is strictly between $R_{c_j}$ and *R*, which is a contradiction to statement 1. or 2., respectively, of Lemma 4.9. The same argument applies to the situations that two unit squares of $R_{C^l_{j}}$ are placed within vertical visibility both above or both below $R_{c_j}$. If there are $R, R' \in R_{C^l_{j}}$ with $R {{\,\mathrm{\downarrow }\,}} R_{c_j}$ and $R_{c_j} {{\,\mathrm{\downarrow }\,}} R'$, they have to be shifted to the same side in order to see each other. However, since *x* and *y* are either aligned or shifted to opposite directions, this means that *R* or $R'$ is strictly between $R_{c_j}$ and *x* or *y*, or *x* or *y* is strictly between *R* or $R'$ and $R_{c_j}$, which is a contradiction to case 1. or 2., respectively, of Lemma 4.9. Consequently, there is at most one $R \in R_{C^l_{j}}$ with $R{{\,\mathrm{\updownarrow }\,}} R_{c_j}$ and, in the following, we assume that $R{{\,\mathrm{\downarrow }\,}} R_{c_j}$ holds (the case $R_{c_j}{{\,\mathrm{\downarrow }\,}} R$ can be handled analogously). In particular, this means *y* is aligned with $R_{c_j}$. Now let $R'$ and $R''$ be the two remaining unit squares from $R_{C^l_{j}}$, i.e., $\{R', R''\} = R_{C^l_{j} \setminus \{c_j\}} \setminus \{R\}$. According to what we observed above, either $R' {{\,\mathrm{\rightarrow }\,}}R_{c_j}$ and $R_{c_j} {{\,\mathrm{\rightarrow }\,}}R''$, or $\{R', R''\} {{\,\mathrm{\rightarrow }\,}}R_{c_j}$. We first assume the former case. According to Lemma 4.5, this means that the $K_4$ on vertices $C^l_{j}$ either satisfies case 1. of Lemma 4.5 with $R_{c_j}$ playing the role of $R_4$ (denote this case as (a)), or case 3. of Lemma 4.5 with $R_{c_j}$ playing the role of $R_3$ (denote this case as (b)):
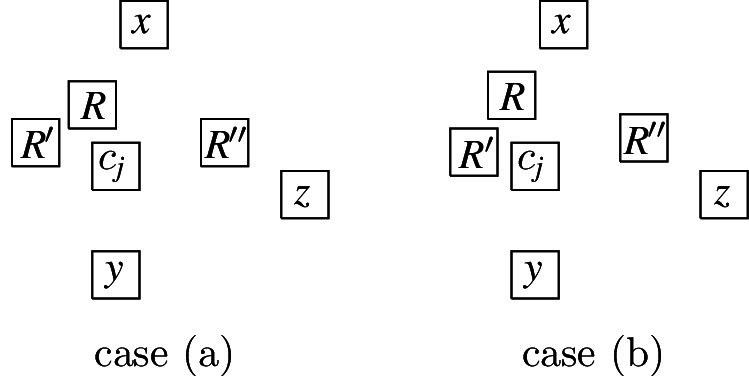
 In both these cases *z* has to be shifted downwards to avoid a contradiction to case 1. or 2. of Lemma 4.9 with $R''$. On the other hand, if $\{R', R''\}{{\,\mathrm{\rightarrow }\,}}R_{c_j}$, then the $K_4$ on $C^l_{j}$ either satisfies case 2. (case (c)), or case 3. of Lemma 4.5 with *R* in the role of $R_4$ (case (d)) or case 3. of Lemma 4.5 with $R'$ in the role of $R_4$ (case (e)):
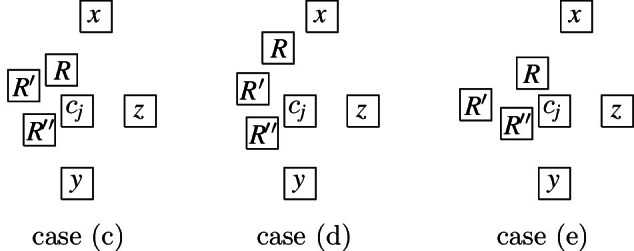
 In these three cases, *z* has to be aligned with $R_{c_j}$ to avoid contradiction with Lemma 4.9. Consequently, under the assumption that, for some $R \in R_{C^l_{j} \setminus \{c_j\}}$, $R {{\,\mathrm{\downarrow }\,}} R_{c_j}$, the unit squares for the $K_4$ on vertices $C^l_{j}$ satisfy one of the cases (a) to (e) illustrated above, and, since the arguments from above apply in the same way, the same holds for the unit squares for the $K_4$ on vertices $C^r_{j}$.
  - Both $K_4$ on vertices $C^l_{j}$ and $C^r_{j}$ satisfy case (a), (b), (c), or (e): We note that this implies that there are $R \in R_{C^l_{j} \setminus \{c_j\}}$ and $S \in R_{C^r_{j} \setminus \{c_j\}}$ with $\{R, S\} {{\,\mathrm{\downarrow }\,}} R_{c_j}$ with a distance of less than one unit from $R_{c_j}$. This is only possible if *R* and *S* are placed (horizontally) next to each other, which means that one of them is strictly between *x* and $R_{c_j}$, which yields a contradiction with case 1. of Lemma 4.9.
  - The $K_4$ on vertices $C^l_{j}$ or the the $K_4$ on vertices $C^r_{j}$ satisfies case (d): We assume that the $K_4$ on vertices $C^l_{j}$ satisfies case (d) (the other case is analogous). We note that this implies that there are $R',R''\in R_{C^l_{j}\setminus \{c_j\}}$ with $\{R', R''\} {{\,\mathrm{\rightarrow }\,}}R_{c_j}$, such that both $R'$ and $R''$ have a horizontal distance of less than one to $R_{c_j}$. This means that there is no $S \in R_{C^r_{j} \setminus \{c_j\}}$ with $S {{\,\mathrm{\rightarrow }\,}}R_{c_j}$ that also has a distance of less that one unit from $R_{c_j}$. Consequently, the $K_4$ on vertices $C^r_{j}$ can only satisfy case (a). However, in this case *z* cannot be aligned with $R_{c_j}$, which contradicts the fact that the $K_4$ on vertices $C^l_{j}$ satisfies case (d) (which requires *z* to be aligned with $R_{c_j}$).
  Consequently, we can assume that there is no $R \in R_{C^l_j \setminus \{c_j\}}$ with $R{{\,\mathrm{\updownarrow }\,}} R_{c_j}$ (or that this holds for the $K_4$ on vertices $C^r_{j}$, which can be handled analogously). Consequently, $R_{C^l_{j} \setminus \{c_j\}} {{\,\mathrm{\leftrightarrow }\,}}R_{c_j}$, which, by Lemma 4.5, implies that either $R_{C^l_{j}\setminus \{c_j\}} {{\,\mathrm{\rightarrow }\,}}R_{c_j}$ or $R_{c_j} {{\,\mathrm{\rightarrow }\,}}R_{C^l_{j} \setminus \{c_j\}}$. Since, as explained above, the latter leads to a contradiction, we can conclude that $R_{C^l_{j} \setminus \{c_j\}} {{\,\mathrm{\rightarrow }\,}}R_{c_j}$. Now if the $K_4$ on vertices $C^r_{j}$ satisfies case (c), (d), or (e), or if this $K_4$ is also realised exclusively by horizontal visibilities, then we obtain a contradiction to Lemma 4.10. Thus, we assume that the $K_4$ on vertices $C^r_{j}$ satisfies case (a) or (b), which means that *z* is not aligned with $R_{c_j}$. This is a contradiction, since, due to Lemmas 4.5 and 4.9, $R_{C^l_{j} \setminus \{c_j\}} {{\,\mathrm{\rightarrow }\,}}R_{c_j}$ implies that *z* must be aligned with $R_{c_j}$.
2. Case 3. ($$\{x, y\} {{\,\mathrm{\downarrow }\,}} R_{c_j}$$ and $$R_{c_j} {{\,\mathrm{\leftrightarrow }\,}}z$$):
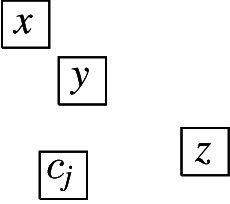
 Due to cases 1. and 2. of Lemma 4.9, we know that neither *x* nor *y* is aligned with $R_{c_j}$ and, furthermore, there exists no $R \in R_{C_{j}}$ with $R {{\,\mathrm{\downarrow }\,}} R_{c_j}$. Next, we assume that there is also no $R \in R_{C_{j}}$ with $R_{c_j}{{\,\mathrm{\downarrow }\,}} R$, which implies $R_{C_j \setminus \{c_j\}} {{\,\mathrm{\leftrightarrow }\,}}R_{c_j}$. By Lemma 4.5, this means that either $R_{C^l_j \setminus \{c_j\}} {{\,\mathrm{\rightarrow }\,}}R_{c_j}$ or $R_{c_j} {{\,\mathrm{\rightarrow }\,}}R_{C^l_j \setminus \{c_j\}}$ and either $R_{C^r_j \setminus \{c_j\}} {{\,\mathrm{\rightarrow }\,}}R_{c_j}$ or $R_{c_j} {{\,\mathrm{\rightarrow }\,}}R_{C^r_j \setminus \{c_j\}}$. However, $R_{c_j} {{\,\mathrm{\rightarrow }\,}}R_{C^l_j \setminus \{c_j\}}$ or $R_{c_j} {{\,\mathrm{\rightarrow }\,}}R_{C^r_j \setminus \{c_j\}}$ yields a contradiction with Lemma 4.9, which implies that $R_{C_{j} \setminus \{c_j\}} {{\,\mathrm{\rightarrow }\,}}R_{c_j}$. This is a contradiction to Lemma 4.10. Consequently, there is at least one $R \in R_{C_{j}}$ with $R_{c_j} {{\,\mathrm{\downarrow }\,}} R$ and, due to case 3. of Lemma 4.9, we can conclude that there is exactly one such unit square that is aligned with $R_{c_j}$. Moreover, without loss of generality, let $R \in R_{C^l_{j}}$. This means that the $K_4$ on vertices $C^l_j$ satisfies case 1. of Lemma 4.5 with $R_{c_j}$ playing the role of $R_2$. In particular, this implies that *z* cannot be aligned with $R_{c_j}$, since this would lead to a contradiction with case 1. or 2. of Lemma 4.9. However, due to the fact $R_{C^r_{j} \setminus \{c_j\}} {{\,\mathrm{\leftrightarrow }\,}}R_{c_j}$, we obtain a contradiction to one of the cases of Lemma 4.9. $\square$

We have now all technical tools at our disposal that are needed to conclude the proof, i.e., to show that the layout for *G* must have the desired structure; thus, it translates into a not-all-equal satisfying assignment for the formula *F*. We first observe that Lemmas 4.9, 4.10, and 4.11 also hold for the part of the graph consisting of the vertices which represent the variables. More precisely, define $X_0=\{c_{m-1},c_{m-1}^1,c_{m-1}^2,c_{m},x_1^1,x_1^2,x_1\}$, $X_1=\{c_{m},x_1^1,x_1^2,x_1,x_2^1,x_2^2,x_2\}$, and $X_i=\{x_{i-1,}x_i^1,x_i^2,x_i,x_{i+1}^1,x_{i+1}^2,x_{i+1}\}$, for all *i*, $2\le i\le n$, and $A_i=\{t_i,f_i^1,f_i^2\}$, for all *i*, $1\le i\le n$. Then Lemma 4.9 also holds for the version where $c_i$ is replaced by $x_i$ and $l^r_i$ is replaced by $t_i$, $f^1_i$, or $f^2_i$, Lemma 4.10 also holds for the version where $C_i$ is replaced by $X_i$ and $c_i$ is replaced by $x_i$ (or $c_{m}$ in case of $X_0$), and Lemma 4.11 also holds for the version where $c_i$ is replaced by $x_i$ and $L_i$ is replaced by $A_i$. This is simply due to the identical structure of these parts of the graph. In the following, we shall refer to these more general versions of the lemmas.

##### Lemma 4.12

If $G \in {{{\,\mathrm{\textsf{USV}}\,}}}$, then *F* is not-all-equal satisfiable.

##### Proof

Let $G \in {{\,\mathrm{\textsf{USV}}\,}}$. By Lemma 4.11 we know that for each *j*, $1\le j\le m-1$, either $R_{c_j}{{\,\mathrm{\leftrightarrow }\,}}R_{L_j}$ or $R_{c_j}{{\,\mathrm{\updownarrow }\,}} R_{L_j}$, so assume that, for some *j*, $1\le j\le m-1$, $R_{c_j}{{\,\mathrm{\updownarrow }\,}} L_{j}$. If, for some $x \in C_{j} \setminus \{c_j\}$, $R_x{{\,\mathrm{\updownarrow }\,}} R_{c_j}$, then we obtain a contradiction with case 1. or 2. of Lemma 4.9; thus, $R_{c_j} {{\,\mathrm{\leftrightarrow }\,}}R_{C_j \setminus \{c_j\}}$. Consequently, for every *j*, $1\le j\le m-1$, $R_{c_j} {{\,\mathrm{\leftrightarrow }\,}}R_{C_j \setminus \{c_j\}}$ or $R_{c_j}{{\,\mathrm{\updownarrow }\,}} R_{C_j \setminus \{c_j\}}$, $R_{c_{m}} {{\,\mathrm{\leftrightarrow }\,}}R_{X_0 \setminus \{c_{m}\}}$ or $R_{c_{m}} {{\,\mathrm{\updownarrow }\,}}R_{X_0 \setminus \{c_{m}\}}$ and, for every *i*, $1\le i\le n$, $R_{x_i} {{\,\mathrm{\leftrightarrow }\,}}R_{X_i \setminus \{x_i\}}$ or $R_{x_i} {{\,\mathrm{\updownarrow }\,}} R_{X_i \setminus \{x_i\}}$.

We assume, without loss of generality, that $R_{c_1} {{\,\mathrm{\leftrightarrow }\,}}R_{C_1 \setminus \{c_1\}}$. By Lemma 4.5, this implies that either $R_{c_1} {{\,\mathrm{\rightarrow }\,}}R_{C^l_1 \setminus \{c_1\}}$ or $R_{C^l_1 \setminus \{c_1\}} {{\,\mathrm{\rightarrow }\,}}R_{c_1}$ and that either $R_{c_1} {{\,\mathrm{\rightarrow }\,}}R_{C^r_1 \setminus \{c_1\}}$ or $R_{C^r_1 \setminus \{c_1\}} {{\,\mathrm{\rightarrow }\,}}R_{c_1}$. Moreover, Lemma 4.10 yields $R_{C^l_1 \setminus \{c_1\}} {{\,\mathrm{\rightarrow }\,}}R_{c_1}$ if and only if $R_{c_1} {{\,\mathrm{\rightarrow }\,}}R_{C^r_1 \setminus \{c_1\}}$. Obviously, this argument applies to every $c_j$, $1 \le j \le m$, and every *i*, $1\le i \le n$. We now assume, without loss of generality, that $R_{C^l_1 \setminus \{c_1\}} {{\,\mathrm{\rightarrow }\,}}R_{c_1}$, which implies $R_{c_1} {{\,\mathrm{\rightarrow }\,}}R_{C^r_1 \setminus \{c_1\}}$ and, in particular, $R_{C^l_2 \setminus \{c_2\}} {{\,\mathrm{\rightarrow }\,}}R_{c_2}$. Repeating this argument inductively on all $C_{j}$, $1\le j\le m-1$, and on all $X_i$, $0 \le i \le n$, implies that the part of the graph consisting of vertices $\{c_i,c_i^1,c_i^2\mid 0\le j\le m-1\}\cup \{c_{m}\}\cup \{x_i,x_i^1,x_i^2\mid 1\le i\le n+1\}$, which we shall call *backbone* in the following, is represented by a layout that is V-isomorphic to the one in Fig. 10, except for the $K_4$ on vertices $R_{C_1^l}$ and the $K_4$ on vertices $x_n, x^1_{n+1}, x^2_{n+1}, x_{n+1}$, which could also satisfy case 3. of Lemma 4.5 (note that all the other $K_4$ must satisfy case 1. of Lemma 4.5, since all their visibilities are horizontal). Moreover, as explained above, this also implies that, for every *j*, $1 \le j \le m$, $R_{L_j}{{\,\mathrm{\updownarrow }\,}} R_{c_j}$ and, for every *i*, $1\le i\le n$, $R_{A_i}{{\,\mathrm{\updownarrow }\,}} R_{x_i}$.

Now, for some *i*, $1 \le i \le n$ and $j_1< j_2<\ldots < j_q$, let $l_{j_1}^{r_1}, \dots , l_{j_q}^{r_q}$ be exactly the selectables corresponding to occurrences of literal $x_i$. By definition, these vertices form a path in this order and the structure of the backbone implies that the *x*-coordinates of their corresponding unit squares differ by at least two, which means that the visibilities between the unit-squares for $l_{j_1}^{r_1},\dots ,l_{j_q}^{r_q}$ are all horizontal; thus, they form a horizontal path in this order and are all on the same side of the backbone. By definition, both vertices $\overset{_{\rightarrow }}{t_i}$ and $\overset{_{\leftarrow }}{t_i}$ are adjacent to all vertices $l_{j_1}^{r_1},\dots ,l_{j_q}^{r_q}$. Since every literal of the formula has at least three occurrences, i.e., $q \ge 3$, the only possibility to place unit squares for $\overset{_{\rightarrow }}{t_i}$ and $\overset{_{\leftarrow }}{t_i}$ in order to see every unit square of the path is horizontally from opposite sides, i.e., either $R_{\overset{_{\rightarrow }}{t_i}} {{\,\mathrm{\rightarrow }\,}}R_{\{l_{j_1}^{r_1}, \dots , l_{j_q}^{r_q}\}}$ and $R_{\{l_{j_1}^{r_1}, \dots , l_{j_q}^{r_q}\}} {{\,\mathrm{\rightarrow }\,}}R_{\overset{_{\leftarrow }}{t_i}}$ or $R_{\overset{_{\leftarrow }}{t_i}} {{\,\mathrm{\rightarrow }\,}}R_{\{l_{j_1}^{r_1}, \dots , l_{j_q}^{r_q}\}}$ and $R_{\{l_{j_1}^{r_1}, \dots , l_{j_q}^{r_q}\}} {{\,\mathrm{\rightarrow }\,}}R_{\overset{_{\rightarrow }}{t_i}}$; since these two cases can be handled analogously, we assume the former. Since the horizontal distance between $R_{\overset{_{\rightarrow }}{t_i}}$ and $R_{\overset{_{\leftarrow }}{t_i}}$ is more than one unit, the unit squares for all other mutual neighbours of $\overset{_{\rightarrow }}{t_i}$ and $\overset{_{\leftarrow }}{t_i}$, i.e., the vertices $h_{t_i}^r$, $0 \le r \le 4$, and $t_i$, must also be placed horizontally in between $R_{\overset{_{\rightarrow }}{t_i}}$ and $R_{\overset{_{\leftarrow }}{t_i}}$. In particular, this implies that $R_{t_i}$ has to be placed on the same side as the path $R_{l_{j_1}^{r_1}},\dots ,R_{l_{j_q}^{r_q}}$ with respect to the backbone.

We define an assignment $\sigma :\{x_1, x_2, \ldots , x_n\} \rightarrow \{true , false \}$ as follows. For every *i*, $1 \le i \le n$, we define $\sigma (x_i) = true$ if and only if $R_{x_i}{{\,\mathrm{\downarrow }\,}} R_{t_i}$. We claim that this assignment is a satisfying not-all-equal assignment for the formula *F*. To this end, let $c_j = \{x_{\ell _1}, x_{\ell _2}, x_{\ell _2}\}$ be an arbitrary clause of *F*. Due to Lemma 4.9 it is not possible that $R_{L_j}{{\,\mathrm{\downarrow }\,}} R_{c_j}$ or $R_{c_j}{{\,\mathrm{\downarrow }\,}} R_{L_j}$, which implies that at least one of $R_{\{t_{\ell _1}, t_{\ell _2}, t_{\ell _3}\}}$ is placed below the backbone and at least one of them is placed above the backbone (since, as explained above, they are placed at the same side as their corresponding unit square from $R_{L_j}$). Consequently, at least one variable occurring in $c_j$ is set to *true* and at least one is set to *false*. $\square$

Lemmas 4.6 and 4.12 show that our reduction is correct. Moreover, it can be easily seen that the reduction can be computed in polynomial time; Consequently, we conclude the following main result of this section:

##### Theorem 4.13

$${{\,\mathrm{\textsc {Rec}}\,}}({{{\,\mathrm{\textsf{USV}}\,}}})$$ is $${{{\,\mathrm{{\textsf{N}}{\textsf{P}}}\,}}}$$-complete.

We conclude this section, by observing that the size of the graph is linear in the size of the formula, which means that we can also conclude ETH-lower bounds for ${{\,\mathrm{\textsc {Rec}}\,}}({{\,\mathrm{\textsf{USV}}\,}})$.

## Conclusions

The hardness of ${{\,\mathrm{\textsc {Rec}}\,}}({{\,\mathrm{\textsf{USV}}\,}}_{{{\,\mathrm{{\textsf{w}}}\,}}})$ is still open (note that in our reduction, we heavily used the argument that certain constellations yield forbidden edges, which falls apart in the weak case) and we conjecture it to be ${{\,\mathrm{{\textsf{N}}{\textsf{P}}}\,}}$-hard as well. Two open problems concerning graph classes related to ${{\,\mathrm{\mathrm {\textsf {USGV}}}\,}}$ are mentioned in Sect. 3: (1) are ${{\,\mathrm{\mathrm {\textsf {USGV}}}\,}}$ and the class of resolution-$(\pi /2)$ graphs identical, (2) are there resolution-$(\pi /2)$ graphs without BRAC drawing? Note that a positive answer to (2) gives a negative answer to (1).

From a parameterised complexity point of view, our ${{\,\mathrm{{\textsf{N}}{\textsf{P}}}\,}}$-completeness result shows that the number of different rectangle shapes (considered as a parameter) has no influence on the hardness of recognition. Another interesting parameter to explore would be the step size of the grid, i.e., for $k \in {\mathbb {N}}$, let ${{\,\mathrm{\mathrm {\textsf {USGV}}}\,}}^k$ be defined like ${{\,\mathrm{\mathrm {\textsf {USGV}}}\,}}$, but for a $\{{\ell }/{k} \,|\,\ell \in {\mathbb {N}}\}^2$ grid. We note that these classes form an infinite hierarchy between ${{\,\mathrm{\mathrm {\textsf {USGV}}}\,}}= {{\,\mathrm{\mathrm {\textsf {USGV}}}\,}}^1$ and ${{\,\mathrm{\textsf{USV}}\,}}= \bigcup _k {{\,\mathrm{\mathrm {\textsf {USGV}}}\,}}^k$, and it is hard to define them in terms of extensions of rectilinear graphs. Another interesting observation is that the hardness reduction for the recognition problem of rectilinear graphs from [19], if interpreted as reduction for ${{\,\mathrm{\textsc {Rec}}\,}}({{\,\mathrm{\mathrm {\textsf {USGV}}}\,}})$, does not work for ${{\,\mathrm{\mathrm {\textsf {USGV}}}\,}}^2$. The classes ${{\,\mathrm{\mathrm {\textsf {USGV}}}\,}}^k$ might be practically more relevant, since placing objects in the plane with discrete distances is more realistic.

Another possible modification of rectangle visibility graphs is that visibility (and therefore adjacency in the represented combinatorial graph) requires also a certain proximity between the (unit) squares. This setting would cater for situations where the components modelled by vertices can only be connected by straight-line segments that meet certain length bounds.

## Figures and Tables

**Fig. 18:**
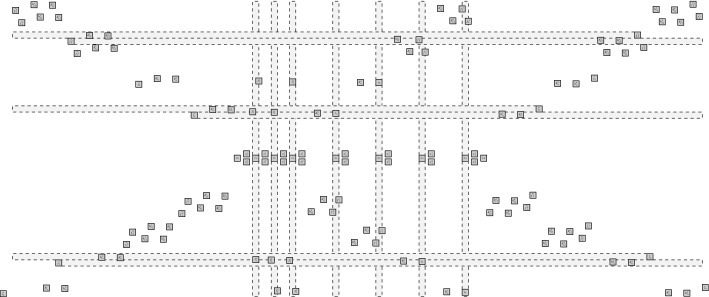
An illustration of the visibility layout for the monotone not-all-equal satisfiable formula $\{c_1, c_2, c_3\}$ with $c_1=\{x_1, x_2, x_3\}$, $c_2 = \{x_1, x_3, x_4\}$, $c_3 = \{x_2, x_3, x_4\}$. The vertical visibilities of the unit squares for the clause vertices $c_j$, $1 \le j \le 3$, and of the unit squares of the variable vertices $x_i$, $1 \le i \le 4$, are highlighted. In addition, the left-to-right horizontal visibilities of the unit squares for the vertices $\overset{_{\rightarrow }}{f_{3}^{2}}$, $\overset{_{\rightarrow }}{t_{1}}$, $\overset{_{\rightarrow }}{t_{3}}$, and the right-to-left horizontal visibilities of the unit squares for the vertices $\overset{_{\leftarrow }}{f_{3}^{2}}$, $\overset{_{\leftarrow }}{t_{1}}$, $\overset{_{\leftarrow }}{t_{3}}$ are highlighted as well (this should also illustrate how the edges between the vertices $\overset{_{\rightarrow }}{t_{i}}, h^1_{t_i}, h^2_{t_i}, l^{q_1}_{j_1}, \ldots , l^{r_q}_{j_q}, h^0_{t_i}, t_i, h^3_{t_i}, h^4_{t_i}, \overset{_{\leftarrow }}{t_{i}}$ (and the corresponding vertex sets including $f^1_{i}$ or $f^2_{i}$) are represented by the layout). The satisfying assignment can be obtained by setting $x_i$ to *true* if and only if $R_{x_i}{{\,\mathrm{\downarrow }\,}} R_{t_i}$, which yields the assignment $x_1 \rightarrow 0$, $x_2 \rightarrow 0$, $x_3 \rightarrow 1$, and $x_4 \rightarrow 1$

## Appendix A

### Details for the Proof of Lemma 4.6

Let *F* be a monotone not-all-equal satisfiable 3-SAT formula with clauses $c_1,\dots ,c_m$ over variables $v_1,\dots , v_n$ and let $\phi :\{x_1\dots ,x_n\}\rightarrow \{0,1\}$ be an according not-all-equal satisfying assignment. The following coordinates yield a ${{\,\mathrm{\textsf{USV}}\,}}$ drawing for the corresponding graph *G* (see Fig. 18 for an illustration). For $j\in \{1,\dots ,m\}$, $h\in \{1,2\}$, $i\in \{1,\dots ,n\}$, $r\in \{1,2,3\}$,

### Example of the Reduction

See Fig. 18 for an illustration of the visibility layout for a monotone Boolean formula with four variables and three clauses.

**Table:**

Vertex	*x*-coordinate	*y*-coordinate
$c_j$	4*j*	0
$c_j^h$	$4j+2$	$2-1.3 h$
$x_i$	$4m+8i$	0
$x_i^h$	$4m+8i-6$	$2-1.3 h$
$t_i$	$4m+8i$	$(-1)^{(1-\phi (x_i))}(5i+2)+1-\displaystyle \frac{2m+4}{2m+8}$
$f_i^h$	$4m+8i+\displaystyle \frac{3}{2}-h$	$(-1)^{\phi (x_i)}(5i+2h)+1-\displaystyle \frac{2m+4}{2m+8}$
$l_j^r$	$4j+\displaystyle \frac{r-k}{2\max {\{1,|r-k|\}}}$	$(-1)^{(1-\phi (x_i))}(5i+2)+1-\displaystyle \frac{j+2}{2m+8}$
	for $y_{j,h}=x_i$, $k={\text {arg\,max}}{\{|r-k|\mid \phi (l_j^r)=\phi (l_j^k)\}}$	
$\overset{_{\rightarrow }}{t_i}$	$-9i$	$(-1)^{(1-\phi (x_i)}(5i+2)$
$\overset{_{\leftarrow }}{t_i}$	$4m+8(n+1)+9i$	$(-1)^{(1-\phi (x_i)}(5i+2) +1$
$\overset{_{\rightarrow }}{f_i^h}$	$-9i-h$	$(-1)^{\phi (x_i)}(5i+2h)$
$\overset{_{\leftarrow }}{f_i^h}$	$4m+8(n+1)+9i-h$	$(-1)^{\phi (x_i)}(5i+2h)+1$
$h^0_{t_i}$	$4m+8i-3$	$(-1)^{(1-\phi (x_i)}(5i+2)+1-\displaystyle \frac{2m+3}{2m+8}$
$h^0_{f_i^h}$	$4m+8i-2h$	$(-1)^{\phi (x_i)}(5i+2h)+1-\displaystyle \frac{2m+3}{2m+8}$
$h^r_{t_i}$	$-9i+3r$	$(-1)^{(1-\phi (x_i)}(5i+2) +1-\displaystyle \frac{r}{2m+8}$
	for $r\in \{1,2\}$	
$h^r_{f_i^h}$	$-9i+3r-h$	$(-1)^{\phi (x_i)}(5i+2h)+1-\displaystyle \frac{r}{2m+8}$
	for $r\in \{1,2\}$	
$h^r_{t_i}$	$4m+8(n+1)+9i+3r-15$	$(-1)^{(1-\phi (x_i)}(5i+2) +\displaystyle \frac{5-r}{2m+8}$
	for $r\in \{3,4\}$	
$h^r_{f_i^h}$	$4m+8(n+1)+9i+3r-h-15$	$(-1)^{\phi (x_i)}(5i+2h)+\displaystyle \frac{5-r}{2m+8}$
	for $r\in \{3,4\}$	

## Footnotes

1 The 24th International Symposium on Graph Drawing and Network Visualization (GD 2016) featured an entire session on visibility representation (see [3, 10, 11, 15]), and the joint workshop day of the Symposium on Computational Geometry (SoCG) and the ACM Symposium on Theory of Computing (STOC) included a workshop on geometric representations of graphs in June 2016.

2 By $\preceq ^{-1}$, we denote the inverse of a binary relation $\preceq$.

3 In the literature (e.g., [17]), the edges of a RAC drawing are usually allowed to have bends; the investigated questions are on finding RAC drawings that minimise the number of bends and crossings. What we call BRAC drawings here, is also denoted as *straight-line* RAC drawings in the literature.

4 Actually, the possibility of touching corners is not explicitly mentioned in [12], but the paper contains example layouts with touching corners that represent graphs that would not be expressible without touching corners (see [12, Fig. 12]).

5 The definition of ${{\,\mathrm{{\textsf{H}}{\textsf{V}}}\,}}$- and ${{\,\mathrm{\textsf{LRDU}}\,}}$-restriction given above naturally translates to rectilinear drawings.

6 Since rectilinear graphs have maximum degree 4, we measure their size in the number of vertices.

7 In general, this space blow-up cannot be avoided, as witnessed by *n* isolated vertices which have a $1\times n$ rectilinear drawing, but a smallest unit square grid layout of size ${(2n-1)}\times {(2n-1)}$.

8 For the more general question of representing bipartite graphs as rectangle visibility graphs, we refer to [14]. In particular, a linear upper bound on the number of edges, compared to the number of vertices, is known.

9 Note that the observations of this paragraph are already covered by the characterisation of trees given in [12]; we merely intend to support the intuitive understanding of unit square visibility graphs by these discussions.
